# Molecular Engineering for Enhanced Thermoelectric Performance of Single‐Walled Carbon Nanotubes/π‐Conjugated Organic Small Molecule Hybrids

**DOI:** 10.1002/advs.202302922

**Published:** 2023-10-20

**Authors:** Tae‐Hoon Kim, Jae Gyu Jang, Sung Hyun Kim, Jong‐In Hong

**Affiliations:** ^1^ Department of Chemistry Seoul National University Seoul 08826 South Korea; ^2^ Department of Carbon Convergence Engineering Wonkwang University Iksan 54538 South Korea

**Keywords:** carbon nanotubes, charge carrier transports, molecular engineering, thermoelectric materials, ZT enhancements, π‐conjugated organic small molecules

## Abstract

Hybridizing single‐walled carbon nanotubes (SWCNTs) with π‐conjugated organic small molecules (π‐OSMs) offers a promising approach for producing high‐performance thermoelectric (TE) materials through the facile optimization of the molecular geometry and energy levels of π‐OSMs. Designing a twisted molecular structure for the π‐OSM with the highest occupied molecular orbital energy level comparable to the valence band of SWCNTs enables effective energy filtering between the two materials. The SWCNTs/twisted π‐OSM hybrid exhibits a high Seebeck coefficient of 110.4 ± 2.6 µV K^−1^, leading to a significantly improved power factor of 2,136 µW m^−1^ K^−2^, which is 2.6 times higher than that of SWCNTs. Moreover, a maximum figure of merit over 0.13 at room temperature is achieved via the efficient TE transport of the SWCNTs/twisted π‐OSM hybrid. The study highlights the promising potential of optimizing molecular engineering of π‐OSMs for hybridization with SWCNTs to create next‐generation, efficient TE materials.

## Graphical abstract

The SWCNTs/π‐conjugated organic small molecule (π‐OSM) hybrid containing a twisted π‐OSM with reduced barrier energy greatly enhanced carrier transport and thermoelectric (TE)properties by maximizing energy filtering (Seebeck coefficient of 113 µV K^−1^). The SWCNTs/twisted π‐OSM achieved the best power factor of 2136 µW m^−1^ K^−2^ and a maximum *ZT* of 0.137 at room temperature among carbon nanotube‐based organic TEs.

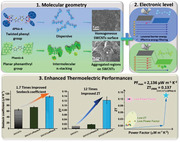

## 1 Introduction

Thermoelectric (TE) materials have emerged as a promising solution for sustainable energy technologies due to their ability to directly convert waste heat into electrical energy, thereby enabling immediate power conversion.^[^ ^1^, ^2^, ^3^ ^]^ The TE efficiency of materials is evaluated by the figure of merit (*ZT*), which can be calculated as *ZT*  =  *S* ^2^ *σT/κ*, where *S*, *σ*, *T*, and *κ* are the Seebeck coefficient, electrical conductivity, absolute temperature, and thermal conductivity, respectively.^[^ ^4^, ^5^, ^6^ ^]^ Hybridization of two different materials is a representative strategy to achieve high *ZT* values by increasing *S* and *σ* and decreasing *κ* through energy filtering, morphological evolution for efficient charge carrier transport, and interfacial phonon scattering, respectively.^[^ ^7^, ^8^ ^]^ Recently, single‐walled carbon nanotubes (SWCNTs)/π‐conjugated organic small molecule (π‐OSM) hybrids have been employed as successful TE hybrid materials, fulfilling the aforementioned strategy. The long‐range sp^2^ carbon network of individual SWCNTs, along with the strong *π*–*π* interactions between SWCNTs, result in excellent *σ*, which in turn leads to a high TE power factor (*PF* = *S^2^σ*). However, SWCNTs have an inherent high *κ*, which poses a challenge in achieving a significant improvement in *ZT*. π‐OSMs can overcome the limitations of SWCNTs as TE materials due to their high *S* and low *κ* as a result of the large bandgap and short‐range order of their sp^2^ carbon network, respectively. Furthermore, the energy levels and molecular geometry of π‐OSMs can be readily adjusted by introducing functional moieties and modifying the length of the π‐conjugated systems using various synthetic methods.^[^ ^9^, ^10^ ^]^ In particular, these unique characteristics enable effective energy filtering and interfacial phonon scattering between the SWCNTs and π‐OSMs in SWCNTs/π‐OSM hybrids, leading to an increase in *S* and a decrease in *κ*. Recently, a pentacenone‐based π‐OSM was incorporated into SWCNTs to create a TE material that achieved a high *PF* of 312 µW m^−1^ K^−2^ by varying the side chains in π‐conjugated moieties.^[^ ^11^ ^]^ Peripheral groups in porphyrin derivatives have been modified to optimize the TE properties in SWCNT/π‐OSM hybrids, resulting in a *PF* of 279.3 µW m^−1^ K^−2^ and an *S* of 53.3 µV K‐1, which is higher than that of neat SWCNTs (38.2 µV K^−1^).^[^ ^12^ ^]^ π‐OSMs with various frameworks have been synthesized and incorporated into SWCNTs/π‐OSM TE hybrids. First, the hybridization of SWCNTs with an amphiphilic π‐OSM composed of a hydrophobic bis(bithiophenyl)‐terphenyl aromatic rod and hydrophilic oligoether chains resulted in simultaneous enhancements of *S* and *σ*, as well as reductions in *κ* through the supramolecular functionalization of the SWCNTs with π‐OSMs.^[^ ^13^ ^]^ Moreover, efficient SWCNTs/π‐OSM TE hybrids were developed by promoting efficient energy filtering through the modulation of the highest occupied molecular orbital (HOMO) level of π‐OSMs relative to the valence band of the SWCNTs.^[^ ^14^ ^]^ Furthermore, the use of an intrinsically twisted π‐OSM, *N*,*N*‐diphenyl‐4′‐(1,2,2‐triphenylvinyl)‐[1,1′‐biphenyl]−4‐amine (TPETPA) in SWCNTs/TPETPA hybrids enabled efficient charge carrier transport, leading to a high *PF* of 539.8 µW m^−1^ K^−2^.^[^ ^15^ ^]^ This increase in *PF* can be attributed to both an increase in *S* and a minimization of the inevitable reduction in *σ*. Previous studies have indicated that achieving effective energy filtering through controlled energy levels of π‐OSM is crucial for systematically improving *S*.^[^ ^14^, ^15^ ^]^ In addition, the molecular geometry of π‐OSM can be a crucial factor in determining the charge carrier transport, which can substantially alter the TE properties such as *S* and *σ*. Therefore, designing π‐OSMs with appropriate molecular geometry and energy levels can enhance the TE properties of SWCNT/π‐OSM hybrids. However, the precise relationship between the molecular shape, energy level, and charge carrier transport in these hybrids remains unclear, necessitating further studies to elucidate their correlation in determining the TE properties.^[^ ^12^, ^16^, ^17^, ^18^ ^]^

This study aims to demonstrate the impact of energy levels and molecular shapes on the TE performance of SWCNTs/π‐OSM TE hybrids. We designed four H‐shaped π‐OSMs with extended aromatic surfaces that incorporate a bithiophene backbone at their core and aryl imidazole groups at both ends, which could be favorable to the charge carrier mobility. Then, we modulated the length of aromatic surfaces of π‐OSMs (**Biz‐6**, **Naphiz‐6**, and **Pheniz‐6**) to investigate how geometrical planarity affects the charge carrier transport and TE properties of SWCNTs/π‐OSMs. Finally, π‐OSMs with twisted phenyl rings (**dPhiz‐6**) were introduced to prevent intermolecular aggregation. Compounds **Biz‐6**, **Naphiz‐6**, and **dPhiz‐6** were identified by ^1^H and ^13^C NMR spectroscopy and high‐resolution mass spectrometry (Figures S1–S4, S6, and S7, Supporting Information). **Pheniz‐6** was characterized by high‐resolution mass spectrometry and ^1^H NMR spectroscopy (Figure S5, Supporting Information). The hybridization of SWCNTs with **dPhiz‐6**, which possesses a twisted geometry and a modulated HOMO level, resulted in a significant improvement in *S* due to maximized energy filtering. In contrast, SWCNTs/**Pheniz‐6**, featuring a flat molecular geometry, exhibited a less pronounced increase in *S* and the lowest *σ*. Moreover, an increase in the hybridization ratio of **dPhiz‐6** in SWCNTs resulted in a significantly reduced *κ*, maximizing the *ZT* values of SWCNTs/**dPhiz‐6**. At a molecular ratio of 32 wt.%, SWCNTs/**dPhiz‐6** exhibited the highest *PF* of 2136 µW m^−1^ K^−2^ and a *ZT* value of over 0.1 at room temperature. The *ZT* value reported in this study is comparable to those of nanostructured SnSe (0.12) and doped PbTe (≈0.10) evaluated at room temperature.^[^ ^19^, ^20^ ^]^

## 2 Results

### 2.1 Material Preparations: π‐OSMs and SWCNTs/π‐OSM Hybrids

**Figure 1:**
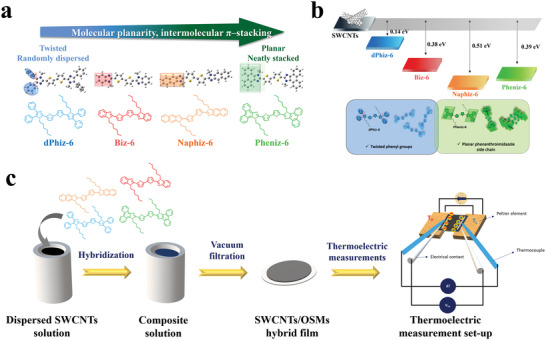
Design strategy of SWCNTs/π‐OSM and preparation of their hybrid films. a) Energy‐minimized molecular structures of **dPhiz‐6, Biz‐6**, **Naphiz‐6**, and **Pheniz‐6**. b) Left: Energy barriers of SWCNTs/**Biz‐6**, SWCNTs/**Naphiz‐6**, SWCNTs/**Pheniz‐6**, and SWCNTs/**dPhiz‐6**. Right: Comparison of the geometrical effect of OSMs on SWCNTs/**Pheniz‐6** and SWCNTs/**dPhiz‐6**. c) SWCNTs/π‐OSM hybrid fabrication process and TE measurement system.

We synthesized π‐OSMs (**Biz‐6**, **Naphiz‐6**, **Pheniz‐6**, and **dPhiz‐6**) by incorporating hexylated arylimidazole derivatives onto both ends of a bithiophene core (**Figure** **1**a). Specifically, **Naphiz‐6** and **Pheniz‐6** were designed to have enlarged planar π surfaces by replacing the benzimidazole moiety of **Biz‐6** with naphthoimidazole and phenanthroimidazole moieties, respectively. In contrast, **dPhiz‐6** was synthesized by incorporating two rotatable phenyl groups at positions 4 and 5 of the imidazole backbone, resulting in a molecular propeller‐like structure. **Biz** and **Naphiz** were prepared via condensation reactions between aryl diamines (1,2‐phenylenediamine and 2,3‐diaminonaphthalene, respectively) and 2,2′‐bithiophene‐5,5′‐dicarboxaldehyde in the presence of *p*‐TsOH. We prepared **Pheniz** and **dPhiz** by conducting one‐pot condensation reactions between the dione (9,10‐phenanthrenedione and benzyl, respectively) and 2,2′‐bithiophene‐5,5′‐dicarboxaldehyde in the presence of ammonium acetate. Bimolecular nucleophilic substitution reactions of 5,5′‐bisarylimidazole‐2,2′‐bithiophene derivatives (**Biz**, **Naphiz**, **Pheniz**, and **dPhiz**) with hexyl iodide under basic conditions yielded *N*‐hexylated π‐OSMs (Scheme S1, Supporting Information). The bandgaps of π‐OSMs were set as the control variable within a range of 2.9–3.0 eV based on the results of density functional theory (DFT) calculations (Figure S8, Supporting Information). The thermal, electronic, and photophysical characteristics of π‐OSMs are reported in the Supporting Information (Table S1 and Figures S9 and S10, Supporting Information). The SWCNTs/π‐OSM hybrids were prepared by mixing a pre‐dispersed SWCNT solution with designated amounts of π‐OSMs (Figure 1c; Figure S11, Supporting Information). The SWCNTs/π‐OSM hybrid solutions were filtered under vacuum and thermally annealed at 100 °C to obtain hybrid films. The energy levels of SWCNTs and π‐OSMs were confirmed through ultraviolet photoelectron spectroscopy (UPS) and CV, and optical spectra (Figure 1b; Figures S12 and S13 and Table S1, Supporting Information). The interfacial barrier energies between SWCNTs and π‐OSMs obtained by UPS are 0.14, 0.38, 0.39, and 0.51 eV for SWCNTs/**dPhiz‐6**, SWCNTs/**Biz‐6**, SWCNTs/**Pheniz‐6**, and SWCNTs/**Naphiz‐6**, respectively. The barrier energy obtained by CV (in solution) exhibited a similar tendency (Figure S13, Supporting Information). Notably, the SWCNTs/**dPhiz‐6** hybrid exhibits the smallest energy barrier, which enables efficient energy filtering and leads to a higher *S*.^[^ ^11^, ^14^, ^18^ ^]^

### 2.2 Confirmation of Hybridizations

**Figure 2:**
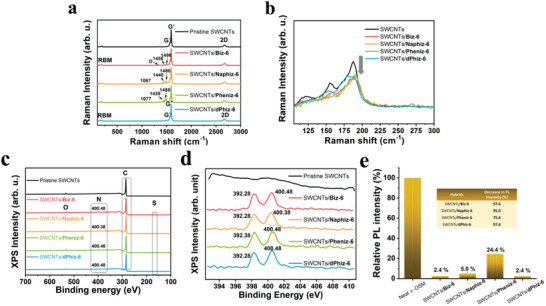
Hybridization of SWCNTs and π‐OSMs. a) Characteristic normal modes and b) radial breathing modes of pristine SWCNTs, SWCNTs/**Biz‐6**, SWCNTs/**Naphiz‐6**, SWCNTs/**Pheniz‐6**, and SWCNTs/**dPhiz‐6** in the Raman spectra. c) Full spectra of X‐ray photoelectron spectroscopy (XPS), d) Observed binding energy of the N‐band in XPS profiles of pristine SWCNTs and the four SWCNTs/π‐OSMs. e) Reduction in photoluminescence (PL) intensity of the four SWCNTs/π‐OSMs compared to that of neat π‐OSMs. Note that the cast masses of the π‐OSMs in the four hybrids are identical to that of neat π‐OSMs.

Raman and photoluminescence (PL) spectra were used to confirm the hybridization of SWCNTs with π‐OSMs. The Raman spectra (**Figure** **2**a) show the characteristic bands of pristine SWCNTs, including 2D (2673 cm^−1^), G^−^, G^+^ (1590 cm^−1^), D (1341 cm^−1^), and radial breathing modes (RBM). The C─H in‐plane bending (1057, 1077 cm^−1^), C─C, and C═C stretching bands (1439, 1440, 1450, 1486, 1488, and 1496 cm^−1^) originated from the bithiophene and aryl imidazole analogs.^[^ ^21^, ^22^ ^]^ The interfacial interactions between SWCNTs and π‐OSMs can influence the vibrational flexibility of SWCNTs, as evidenced by the comparison between RBM band intensities of SWCNTs/π‐OSM and pristine SWCNTs.^[^ ^23^, ^24^ ^]^ The decrease in RBM intensity observed in the SWCNTs/π‐OSMs hybrids compared to that of pristine SWCNTs (Figure 2b), which is attributed to disturbed oscillation in the radial direction of SWCNTs due to robust *π*–*π* interactions between the SWCNTs and π‐OSMs, provides evidence of successful hybridization between SWCNTs and π‐OSMs. Further confirmation of the hybridization between SWCNTs and π‐OSMs was obtained through X‐ray photoelectron spectroscopy (XPS) and PL analyses. The XPS profiles showed characteristic peaks in the ranges of 160–169 eV and 395–405 eV, which correspond to the sulfur of the bithiophene backbones and nitrogen of the imidazole in the π‐OSMs, respectively, indicating the presence of π‐OSMs in SWCNTs/π‐OSMs (Figure 2c). In addition, the XPS band of nitrogen shifted to higher binding energy upon hybridization with SWCNTs, indicating charge transfer from the π‐OSM to the SWCNTs.^[^ ^25^, ^26^ ^]^ The magnified nitrogen band in the range of 394–410 eV clearly indicated the peaks originated from aryl imidazole scaffolds, whereas pristine SWCNTs did not show any signals for the nitrogen band (Figure 2d). In SWCNTs/organic semiconductor hybrid materials, the degree of PL quenching serves as a useful indicator for monitoring molecular adsorption and supramolecular interactions.^[^ ^27^, ^28^ ^]^ The PL spectra of SWCNTs/**Biz‐6** and SWCNTs/**dPhiz‐6** exhibited almost complete quenching, with quenching efficiencies of 97.6% for both hybrids (Figure 2e; Figure S14a,d, Supporting Information). The PL quenching efficiency of SWCNTs/**Naphiz‐6** was slightly lower at 95.0% (Figure S14b, Supporting Information). In contrast, SWCNTs/**Pheniz‐6** exhibited lower PL quenching than the other hybrid films, with a quenching efficiency of 75.6% (Figure S14c, Supporting Information). The low PL quenching and adsorption observed in the SWCNTs/**Pheniz‐6** hybrid suggest a weak interaction between SWCNTs and **Pheniz‐6**, which could potentially result in inhomogeneity and adversely affect the TE performance.

### 2.3 TE Properties of SWCNTs/π‐OSM Hybrids

**Figure 3:**
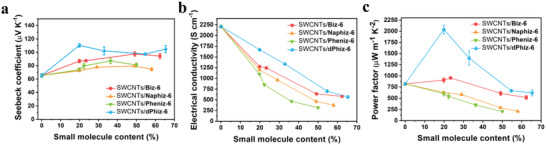
TE properties of SWCNTs/π‐OSM hybrids. a) Seebeck coefficients *S*, b) Electrical conductivities *σ*, and c) Power factors (*PF)* of SWCNTs/**Biz‐6**, SWCNTs/**Naphiz‐6**, SWCNTs/**Pheniz‐6**, and SWCNTs/**dPhiz‐6**. The adsorption amounts of the SWCNTs/π‐OSMs were calculated using UV–vis quantitative analysis (Figure S15 and Table S2, Supporting Information).

The *σ* of the four hybrid films exhibited a decreasing trend with increasing ratios of hybridized π‐OSMs (**Figure** **3**b). This could be attributed to the energy filtering and charge‐carrier scattering mechanisms that occur at the interfaces between SWCNTs and π‐OSMs. Specifically, the *σ* of SWCNTs/**Pheniz‐6** showed a significant reduction when increasing the π‐OSM molecular ratio from 19 to 22 wt.%, followed by a continued decline at ratios above 22 wt.%. Among the four hybrids, SWCNTs/**dPhiz‐6** exhibited the highest *σ* values (1671.4 ± 0.2–701.6 ± 35.1 S cm^−1^) in the molecular content range of 19–58 wt.%, followed by SWCNTs/**Biz‐6** (905.5 ± 52.1–608.0 ± 41.8 S cm^−1^), SWCNTs/**Naphiz‐6** (627.8 ± 10.4–207.0 ± 11.9 S cm^−1^), and SWCNTs/**Pheniz‐6** (602.6 ± 44.1–207.4 ± 15.0 S cm^−1^). The *S* values of the hybrids (Figure 3a) were found to be related to their HOMO energy levels. Among the hybrids, SWCNTs/**dPhiz‐6** exhibited a plateau region (98–110 µV K^−1^) in the region of a molecular content of 19–65 wt.%. The *S* of SWCNTs/**Naphiz‐6** and SWCNTs/**Pheniz‐6** decreased beyond a molecular content of 40 wt.%. Notably, SWCNTs/**dPhiz‐6**, with the lowest barrier energy (0.14 eV), exhibited the highest *S* (110.4 ± 2.6 µV K^−1^) at a molecular content of 19 wt.% among all molecular ratios, followed by SWCNTs/**Biz‐6** (86.8 ± 2.4 µV K^−1^) with a barrier energy of 0.38 eV, SWCNTs/**Pheniz‐6** (73.9 ± 2.7 µV K^−1^) with a barrier energy of 0.39 eV, and SWCNTs/**Naphiz‐6** (72.3 ± 0.5 µV K^−1^) with a barrier energy of 0.51 eV. As observed in inorganic composites, the HOMO energy level difference between the SWCNTs and **dPhiz‐6** was sufficiently low to induce preferential energy‐dependent charge carrier scattering,^[^ ^29^, ^30^, ^31^ ^]^ which led to the largest increase in the *S*, as observed in the SWCNTs/**dPhiz‐6** hybrid.^[^ ^14^, ^15^, ^32^ ^]^ The *PFs* of 19 and 23 wt.% SWCNTs/**Biz‐6** (905.5 ± 52.1 and 953.0 ± 26.1 µW m^−1^ K^−2^, respectively) were slightly higher than those of pristine SWCNTs (820.3 ± 3.2 µW m^−1^ K^−2^). On the contrary, the highest increase in the *S* observed in the SWCNT/**dPhiz‐6** hybrid resulted in a notable enhancement of its *PF* to 2038.4 ± 97.0 µW m^−1^ K^−2^, the best among all SWCNTs/organic molecule TE hybrids studied (Figure 3c; Table S3, Supporting Information). TE properties of SWCNTs/**Biz**, SWCNTs/**Naphiz**, and SWCNTs/**dPhiz** (**Biz**, **Naphiz**, and **dPhiz** without the hexyl groups on the *N*‐terminal of the imidazole moiety, named “precursors” of **Biz‐6**, **Naphiz‐6**, and **dPhiz‐6**) were also investigated (Table S4, Supporting Information). Similar to the trend observed for SWCNTs/π‐OSMs, the highest TE performance (*PF*) among SWCNTs/precursor hybrids was achieved in SWCNTs/**dPhiz** with 20 wt.% of **dPhiz** mixing ratio (320.1 ± 27.7 µW m^−1^ K^−2^), followed by SWCNTs/**Biz** (164.6 ± 28.6 µW m^−1^ K^−2^) and SWCNTs/**Naphiz** (111.8 ± 23.2 µW m^−1^ K^−2^).

### 2.4 TE Transport Properties

**Figure 4:**
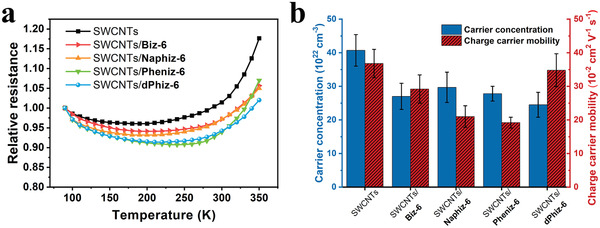
Hall‐effect measurements and temperature dependence of charge carrier transport. a) Normalized resistances of SWCNTs and SWCNTs/π‐OSM (90−350 K) with 20 wt.% mixing ratios. b) Carrier concentrations and charge carrier mobilities of SWCNTs, SWCNTs/Biz‐6, SWCNTs/Naphiz‐6, SWCNTs/Pheniz‐6, and SWCNTs/dPhiz‐6.

Temperature‐dependent resistance and Hall‐effect measurements (as shown in **Figure** **4**) were performed to investigate the effect of the HOMO level differences among the given π‐OSMs on the electrical and TE transport properties of SWCNTs/π‐OSMs.^[^ ^33^ ^]^ The temperature‐dependent resistances were measured in the range 90–350 K (Figure 4a). The relative resistance curves of both pristine SWCNTs and SWCNTs/π‐OSM hybrids were fitted to the heterogeneous model (Figure S16, Supporting Information; see Equation (1)),^[^ ^34^ ^]^ where *T*_m_ and *T*_s_ are characteristic temperatures for metallic and semiconducting conduction, *R*_m_ and *R*_s_ are metallic and semiconducting resistances, *D* is the dimension, and *α* and *β* are geometric factors that correspond to the metallic and semiconducting (variable range hopping) character.^[^ ^35^, ^36^ ^]^

(1) $$R\;\left(T\right)=\alpha R_{m}e^{-\frac{T_{m}}{T}}+\beta R_{s}e^{{\left(\frac{T_{s}}{T}\right)}^{\left(\frac{1}{D+1}\right)}}$$

The pristine SWCNTs displayed the strongest metallic conduction above 200 K, as indicated by the largest α*R_m_*‐value (335.5) (Table S5, Supporting Information). Notably, the value of α*R_m_* (9.8) for SWCNTs/**dPhiz‐6** (Table S5, Supporting Information) was significantly smaller than those of SWCNTs/**Biz‐6**, SWCNTs/**Naphiz‐6**, and SWCNTs/**Pheniz‐6** (26.9, 25.2, and 283.0, respectively), implying that the semiconducting conduction in SWCNTs**/dPhiz‐6** was much more dominant than in other hybrids. According to the heterogeneous conduction model, the conduction behavior of metal‐like SWCNT bundles can be altered by less‐conductive contact junctions between SWCNT bundles, eliciting the emergence of both metallic and semiconducting portions. In the SWCNTs/π‐OSM hybrid system, charge carriers must traverse the more conductive SWCNT bundles while overcoming the barrier posed by the less conductive π‐OSMs in order to transmit the current flow.^[^ ^35^, ^36^, ^37^ ^]^ Therefore, π‐OSMs with HOMO energy levels that differ from those of SWCNTs can increase variable hopping distances, leading to decreased α*R_m_* and increased β*R_s_* values in the SWCNTs/π‐OSM hybrid.^[^ ^33^ ^]^ To further explore the intrinsic carrier transport properties of SWCNTs/π‐OSM hybrids, we conducted Hall‐effect measurements (Figure 4b; Table S6, Supporting Information). The lower the energy barrier in the SWCNTs/π‐OSM hybrid, the more favorable was the charge carrier transport between SWCNTs and π‐OSMs. These aspects accelerated the charge carrier transport between SWCNTs and **dPhiz‐6** (*E*_B_  =  0.14 eV), resulting in a more pronounced enhancement of *S* compared to the other hybrids. In this context, the occurrence of energy filtering can lead to a reduced charge carrier concentration. The charge carrier concentration of SWCNTs/**dPhiz‐6** (2.5 ± 0.3 × 10^22^ cm^−3^) is relatively smaller than those of SWCNTs/**Biz‐6** (2.7 ± 0.3 × 10^22^ cm^−3^), SWCNTs/**Naphiz‐6** (3.0 ± 0.4 × 10^22^ cm^−3^), and SWCNTs/**Pheniz‐6** (2.8 ± 0.2 × 10^22^ cm^−3^), implying that effective carrier transport occurs between SWCNTs and **dPhiz‐6**. The charge carrier mobilities of the three hybrid systems except for SWCNTs/**dPhiz‐6** showed a significant decrease, ranging from 1.9 × 10^−1^ to 2.9 × 10^−1^ cm^2^ V^−1^ s^−1^, whereas the charge carrier mobility of SWCNTs/**dPhiz‐6** (3.5 ± 0.4 × 10^−1^ cm^2^ V^−1^ s^−1^) was found to be similar to that of pristine SWCNTs (3.7 ± 0.4 × 10^−1^ cm^2^ V^−1^ s^−1^). The elevated charge carrier mobility of SWCNT/**dPhiz‐6** was the primary factor contributing to the sustained highest *σ* amongst the hybrid systems.

### 2.5 Morphological Studies

**Figure 5:**
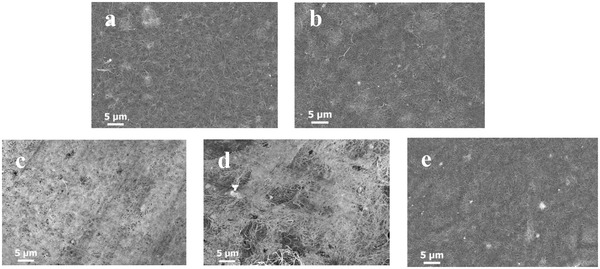
SEM images of a) pristine SWCNTs, b) SWCNTs**/Biz‐6** (62 wt.%), c) SWCNTs**/Naphiz‐6** (58 wt.%), d) SWCNTs/**Pheniz‐6** (50 wt.%), and e) SWCNTs/**dPhiz‐6** (65 wt.%).

To clarify the impact of various morphologies on the charge carrier mobilities and electrical properties of SWCNTs/π‐OSMs, we conducted scanning electron microscopy (SEM), electron probe microanalysis (EPMA), and Raman mapping. The main purpose was to establish a definitive correlation between the aggregation of π‐OSMs and the TE performance of SWCNTs/π‐OSM hybrids.^[^ ^17^, ^38^ ^]^ SEM surface images of both pristine SWCNTs and hybrid films (each with 80 wt.% π‐OSM content) revealed two distinct features. As shown in **Figure** **5**b,e, SWCNTs/**Biz‐6** and SWCNTs/**dPhiz‐6**, respectively, exhibited relatively uniform carbon nanotube networks that were comparable to those of pristine SWCNTs. The images at even lower molecular concentrations (Figure S17c,d, Supporting Information) also revealed a stark contrast in the morphologies of SWCNTs/**Pheniz‐6** and SWCNTs/**dPhiz‐6**, which arose from the distinct molecular structures between **Pheniz‐6** and **dPhiz‐6**. EPMA images of four SWCNTs/π‐OSMs (Figure S18b–e, Supporting Information) mapped by Sulfur (S) showed uniform distributions of elemental *S* which originated from the common bithiophene backbone in π‐OSMs, whereas that of SWCNTs (Figure S18a, Supporting Information) exhibited no signal of *S*. This clear contrast elicits evidence for the existence of π‐OSMs on the surface of the SWCNTs hybrid. Raman mapping results for SWCNTs/**Naphiz‐6** and SWCNTs/**Pheniz‐6** (Figure S19b,c, Supporting Information) revealed distinct red domains characterized by intense C═C stretching over the G band. These domains originated from the bithiophene backbone within the π‐OSMs. On the contrary, the Raman mapping images of SWCNTs/**dPhiz‐6** and SWCNTs/**Biz‐6** (Figure S19a,d, Supporting Information) displayed evenly green surfaces with a low intensity of C═C stretching. This suggests the presence of molecularly aggregated domains of **Naphiz‐6** and **Pheniz‐6** within SWCNTs/**Naphiz‐6** and SWCNTs/**Pheniz‐6**, probably due to their planar molecular structures, which can serve as charge scattering centers and lower charge carrier mobilities (Figure S14c,g, Supporting Information). As a result, this led to much lower charge carrier transport efficiency of SWCNTs/**Pheniz‐6** compared to that of SWCNTs/**dPhiz‐6,** which exhibits higher carrier mobility and *S* (Table S6, Supporting Information; Figure 4b).

### 2.6 Mechanistic Considerations

**Figure 6:**
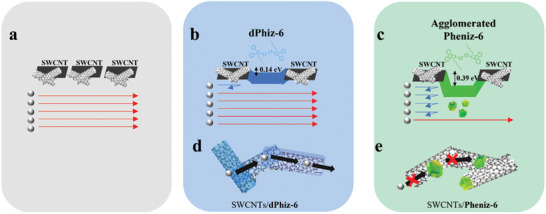
Suggested charge carrier transport behaviors. Charge carrier movements in a) pristine SWCNTs, b) SWCNT–**dPhiz‐6**–SWCNT junctions, and c) SWCNT–**Pheniz‐6**–SWCNT junctions according to energy level differences. The charge carrier transport behaviors in d) SWCNTs/**dPhiz‐6** and e) SWCNTs/**Pheniz‐6** hybrid materials.

**Figure 7:**
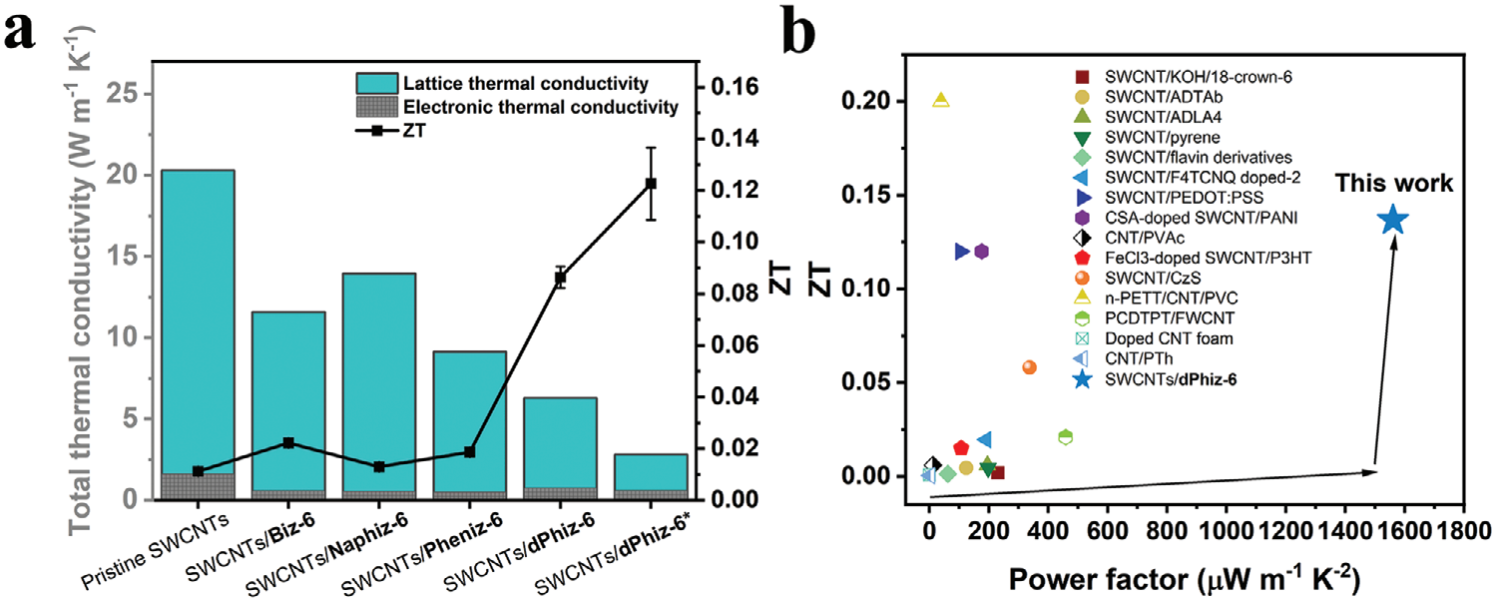
High‐performance SWCNTs/π‐OSM hybrids as TE materials. a) Thermal conductivities and *ZT* values of pristine SWCNTs, SWCNTs/**Biz‐6** (19 wt.%), SWCNTs/**Naphiz‐6** (19 wt.%), SWCNTs/**Pheniz‐6** (19 wt.%), SWCNTs/**dPhiz‐6** (19 wt.%), and SWCNTs/**dPhiz‐6** (32 wt.%). b) Comparison with TE performances of previously reported carbon nanotube‐based organic hybrid materials.

Based on our results, we propose the following charge carrier transport mechanisms in the SWCNTs/π‐OSM hybrids (**Figure** **6**). First, the charge carrier transport in the hybrids is significantly influenced by the difference in HOMO levels between the SWCNTs and π‐OSMs. There are two possible charge carrier transport pathways in the hybrids: SWCNTs–SWCNTs–SWCNTs and SWCNTs–π‐OSMs–SWCNTs. In the case of SWCNTs/**dPhiz‐6**, where the HOMO level of **dPhiz‐6** is similar to that of SWCNTs (*E* **_B_**  =  0.14 eV), the primary pathway for charge carrier transport is through SWCNTs–**dPhiz‐6**–SWCNTs, whereas for SWCNTs/**Pheniz‐6**, the charge carrier transport through the SWCNTs–**Pheniz‐6**–SWCNTs pathway is less dominant compared to SWCNTs–SWCNTs–SWCNTs (Figure 6a) due to the much higher energy barrier of SWCNTs/**Pheniz‐6** (*E* **_B_**  = 0.39 eV). As a result, the energy filtering process via SWCNTs–**dPhiz‐6**–SWCNTs (Figure 6b) was facilitated much more than that through SWCNTs–**Pheniz‐6**–SWCNTs (Figure 6c), leading to the higher *S* of SWCNTs/**dPhiz‐6** compared to SWCNTs/**Pheniz‐6**. Second, the morphological effect induced by the inherent molecular geometry could result in different *σ* between SWCNTs/**dPhiz‐6** and SWCNTs/**Pheniz‐6**. The twisted geometry of **dPhiz‐6** prevented the formation of aggregates even with an increase in molecular content, whereas the planar structure of **Pheniz‐6** easily triggered agglomeration in SWCNT networks by intermolecular π‐stacking. The aggregation of SWCNTs**/Pheniz‐6** generated numerous charges scattering sites, significantly reducing the charge carrier mobility of SWCNTs**/Pheniz‐6,** as shown in Figure 4b. The aggregation‐induced scattering of charge carriers in SWCNTs/**Pheniz‐6** presumably offsets the increase in *S* induced by the energy filtering effect, thereby hampering any noticeable enhancement in *S*. Therefore, charge carriers in SWCNTs/**dPhiz‐6** could pass along shallow barriers through SWCNTs–**dPhiz‐6**–SWCNTs (Figure 6d) based on the hopping mechanism, whereas in SWCNTs/**Pheniz‐6**, charge carriers predominantly move through SWCNT–SWCNT–SWCNT (Figure 6a) due to the steep barrier energy and the prevalent aggregated domains (Figure 6e). This explanation is supported by the observation of high metallic resistance in the SWCNTs**/Pheniz‐6** hybrid. Considering the origin of their distinct transport characteristics, it can be concluded that SWCNTs**/Pheniz‐6**, with poor surface homogeneity and inefficient charge carrier movement, exhibited a severely deteriorated *σ* and TE performance. However, the effective charge carrier transport in SWCNTs/**dPhiz‐6** resulted in significantly higher carrier mobility compared to that of SWCNTs**/Pheniz‐6**, leading to the highest *σ* among the four hybrid materials. The advantageous morphology and energy levels of SWCNTs/**dPhiz‐6** led to a more semiconducting‐like charge carrier transport, resulting in improved *PF* and *ZT* values (**Figure** **7**).

### 2.7 Thermal Conductivities and ZTs of SWCNTs/π‐OSM Hybrids

The total thermal conductivities (*κ*_Total_) of SWCNTs/π‐OSM hybrids were calculated using the formula *κ*_Total_ *=*  *αρC*_p_ (Table S6, Supporting Information) to evaluate *ZT*s, where *α, ρ*, and *C*_p_ refer to the thermal diffusivity, density, and specific heat capacity, respectively.^[^ ^39^ ^]^ The values for *κ*_Total_, lattice thermal conductivity (*κ*_L_), and electronic thermal conductivity (*κ*_E_) were estimated using Equations (2) and (3), where *L* is the Lorenz number, equivalent to 1.5 × 10^−8^ V^2^ K^−2^ for organic semiconductors.^[^ ^13^, ^19^, ^40^ ^]^

(2) $$\kappa_{E}=L\sigma T$$

(3) $$\kappa_{Total}=\kappa_{L}+\kappa_{E}$$

The thermal diffusivities were measured in the in‐plane direction using the Ångström method. The *κ*_L_ of the SWCNTs and hybrids accounted for most of the *κ*_Total_ (Figure 7a). The *κ*_E_ values of the hybrids (0.49–0.75 W m^−1^ K^−1^) were found to be slightly lower than that of the SWCNTs (1.60 W m^−1^ K^−1^). The *κ*_L_ values of SWCNTs/**Biz‐6** (19 wt.%) and SWCNTs/**Naphiz‐6** (19 wt.%) (11.58 and 13.95 W m^−1^ K^−1^, respectively) were decreased compared to that of SWCNTs (20.30 W m^−1^ K^−1^), while that of SWCNTs/**dPhiz‐6** (19 wt.%) was remarkably reduced to 6.28 W m^−1^ K^−1^. The high *κ*_Total_ of the pristine SWCNTs resulted in a *ZT* value of 0.011. However, upon the incorporation of 19 wt.% π‐OSMs in the SWCNTs, there was a notable reduction in *κ*_Total_, which in turn led to a gradual increase in the *ZT* values, ranging from 0.013 to 0.022. Remarkably, the maximum *ZT* value of the SWCNTs/**dPhiz‐6** hybrid (0.0905) exhibited an 810% increase compared to that of the pristine SWCNTs. When 32 wt.% of **dPhiz‐6** was incorporated into the SWCNTs‐based hybrid, there was a significant 84% reduction in *κ*_Total_ (3.40 W m^−1^ K^−1^) as compared to the pristine SWCNTs (21.90 W m^−1^ K^−1^), resulting in a remarkable increase in the *ZT* value of the SWCNTs/**dPhiz‐6** hybrid to 0.123 ± 0.014 at room temperature, 1220% higher than that of the pristine SWCNTs. By incorporating a suitably designed **dPhiz‐6** into SWCNTs, the resulting SWCNTs/**dPhiz‐6** hybrid exhibited a remarkably high *ZT* value. Moreover, its *PF* is the highest reported among organic TE materials hybridized with carbon nanotubes to date (Table S7, Supporting Information).^[^ ^12^, ^13^, ^33^, ^41^, ^42^, ^43^, ^44^, ^45^, ^46^, ^47^, ^48^, ^49^, ^50^, ^51^, ^52^, ^53^, ^54^, ^55^ ^]^ The maximum *PF* of 2136 µW m^−1^ K^−2^ and *ZT* value of 0.137 at 298 K were achieved at **dPhiz‐6** ratios of 19 and 32 wt.%, respectively. The ultrahigh *PF* observed in the SWCNTs/**dPhiz‐6** hybrid is attributed to the efficient charge carrier transport enabled by energy filtering between the SWCNTs and π‐OSM. Furthermore, a noticeable reduction in the *κ*_Total_ of SWCNTs**/dPhiz‐6** resulted in a significant enhancement of its *ZT* value. The simultaneous achievement of high *PF* and *ZT* in this study provides a great advantage for TE power generation. Remarkably, the TE performance of the SWCNTs/**dPhiz‐6** hybrid material surpassed those reported in previous studies, even in the absence of additional carrier injections by molecular dopants (Figure 7b; Table S7, Supporting Information). These results provide valuable molecularly guided insights into the rational construction of organic hybrid materials for achieving high‐performance TE applications. Assuming that different constituents in hybrid‐based materials possess similar HOMO energy levels, the energy‐dependent scattering of charge carriers could improve the *S* of the hybrid material.^[^ ^31^, ^32^, ^56^ ^]^ The SWCNTs/**dPhiz‐6** hybrid system displayed a barrier energy of 0.14 eV, which is low enough to facilitate energy filtering at the SWCNTs–**dPhiz‐6** interfaces, leading to a remarkable improvement in the *S* value (110.4 ± 2.6 µV K^−1^) compared to that of pristine SWCNTs (65.4 ± 2.6 µV K^−1^). In contrast, the *S* of SWCNTs/**Pheniz‐6** (73.9 ± 2.7 µV K^−1^) exhibited only a slight increase, presumably due to a relatively inefficient energy filtering effect of SWCNTs/**Pheniz‐6**. The disparity in *S* values between SWCNTs/**Pheniz‐6** and SWCNTs/**dPhiz‐6** can be attributed to the significant charge‐carrier scattering in the former, which impeded the efficient energy filtering and subsequent enhancement of *S*. The superiority of SWCNTs/**dPhiz‐6** over SWCNTs/**Pheniz‐6** in terms of π‐π interactions and carrier transport properties was reconfirmed through PL and adsorption experiments on the hybrid materials (Figure 2d; Table S2, Supporting Information). When considering the TE performance of SWCNTs‐based hybrid materials, the twisted‐shaped **dPhiz‐6** molecules in SWCNTs/**dPhiz‐6** exhibited greater advantages than the planar‐shaped **Pheniz‐6** molecules in SWCNTs/**Pheniz‐6**. These findings highlight the importance of optimizing the molecular design of the organic species for efficient charge transport in hybrid materials for enhanced TE performance.

### 2.8 Flexibility and Environmental Durability Test

Given the crucial importance of long‐term TE stability under both air exposure and bending conditions for practical outdoor applications, we conducted comprehensive testing to assess the endurance of SWCNTs**/dPhiz‐6** in terms of the air stability and durability (Figure S20 and Table S9, Supporting Information). The normalized Seebeck coefficients (*S/S*_0_) and electrical conductivities (*σ/σ*_0_) of SWCNTs/**dPhiz‐6** retained a remarkable 94 and 98% of their initial TE properties, respectively, even after undergoing harsh bending cycles of 1000 times (Figure S20, Supporting Information). Air stability of SWCNTs/**dPhiz‐6** was tested as it exhibited the greatest TE performance. Under the conditions of air exposure for a period exceeding 3 weeks, the initial values of *S*_0_ and *σ*_0_ for SWCNTs/**dPhiz‐6** displayed a minor decrease of 3% and 1%, respectively, attesting to its outstanding ambient stability (Table S9, Supporting Information).

### 2.9 Application to TE Generators Utilizing SWCNTs/π‐OSM Hybrids

**Figure 8:**
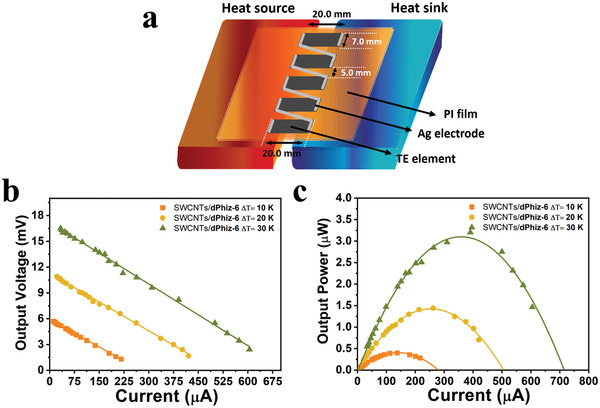
a) Illustrated structure of the flexible TE module with electrically connected TE p‐legs. Voltage–current output curves of b) SWCNTs/**dPhiz‐6** and c) power–current output of SWCNTs/**dPhiz‐6** obtained from TE modules at different temperature gradients.

A prototype TE module for power generation was implemented employing our SWCNTs/**dPhiz‐6**‐based TE materials. Five rectangular‐shaped TE legs embedded onto a polyimide (PI) substrate were serially interconnected by zigzag patterns of Ag electrodes. Illustrations of our TE module with a customized geometry of five p‐type legs are presented in **Figure** **8**a. The power–current output and voltage‐current output curves of the TE modules were recorded at the identical temperature gradients of 10, 20, and 30 K. As the temperature gradients (*ΔT*) increased, both the voltage and current of SWCNTs/**dPhiz‐6** also proportionally increased, with the highest short‐circuit current of 605 µA and the peak open‐circuit voltage (*V*_OC_) reaching up to 16.5 mV (Figure 8b).

Figure 8c showed a maximum output power of 3.2 µW for SWCNTs/**dPhiz‐6**, which was greatly larger than that of SWCNTs (Figure S21, Supporting Information). Compared to other reported TE modules fabricated in a similar geometry, the output powers of SWCNTs/**dPhiz‐6** were superior at various temperature gradients.^[^ ^11^ ^]^ This indicates that our TE materials of molecularly‐engineered SWCNTs/**dPhiz‐6** could be a promising candidate for future TE power generations.

## 3 Discussion

First, we successfully identified the effect of energy levels associated with the π‐OSMs (**Biz‐6**, **Naphiz‐6**, **Pheniz‐6**, and **dPhiz‐6**) on the *S* of the hybrid SWCNTs/π‐OSMs material. Energy levels of the π‐OSMs and the SWCNTs/π‐OSMs hybrid were thoroughly investigated by various techniques, including theoretical quantum calculations, UV–vis and photoluminescence spectroscopy, ultraviolet photoelectron spectroscopy, and cyclic voltammetry.

Second, we demonstrated that the design of a twisted‐shaped π‐OSM, possessing a HOMO energy level similar to that of SWCNTs, is an attractive way for achieving a substantial performance enhancement in organic TE materials based on SWCNT/π‐OSM hybrids. The favorable interaction between the twisted π‐OSM and SWCNTs selectively hinders intermolecular π‐stacking within the twisted π‐OSM, thereby maintaining the *σ*. The greatly improved *S* coupled with the high *σ* elicited the highest *PF* of 2136 µW m^−1^ K^−2^ and a *ZT* value of 0.137 among carbon nanotube‐based organic TE materials.

Third, we established a direct link between the distinctive charge carrier transport behavior and the elevation in the *S* through the energy filtering effect. This enabled us to provide a logical interpretation for the enhancement of TE performances through the assistance of a molecular perspective.

Lastly, we successfully adapted SWCNTs/π‐OSMs materials into prototype TE modules, paving the way for efficient power generation. Witnessing the flexibility and durability demonstrated by our materials, we believe that our strategy based on molecular engineering could be potentially applicable to the development of high‐performance and enduring TE devices for sustainable energy harvesting.

In summary, we demonstrated that strategically engineering twisted π‐OSMs with energy levels comparable to those of SWCNTs creates an optimal SWCNTs/π‐OSM system that enables effective energy filtering, resulting in a substantial enhancement of the TE performance of SWCNTs‐hybrid materials. Integration of twisted **dPhiz‐6** with SWCNTs (SWCNTs/**dPhiz‐6**) successfully prevented aggregation of carbon nanotube nanostructures, leading to a high *ZT*, exceeding 0.13, and the highest maximum *PF* (2136 µW m^−1^ K^−2^) among reported organic hybrid materials containing carbon nanotubes at room temperature. Our study revealed that by employing an appropriate molecular design of π‐OSMs, excellent TE efficiency can be achieved without resorting to additional doping processes. The systematic molecular‐level approach proposed in this work offers a valuable platform for future development of high‐performance organic hybrid TE devices, providing several advantages including ease of fabrication, flexibility, and durability.

## 4 Experimental Section

### Reagents and Materials

Unless otherwise noted, all solvents and reagents were utilized without further purification. SWCNTs (93% purity, OCSiAl Asia Pacific Co., Ltd.) were utilized to prepare SWCNTs/π‐OSMs hybrid materials.

### Spectroscopic, Electronic, and Thermal Studies

Quantum calculations were performed using the DFT/ B3LYP method (Gaussian 09) with the 6–31G* basis set. ^1^H and ^13^C NMR spectra were acquired on a Varian 500 MHz spectrometer (Varian, Inc.) using DMSO‐*d*_6_ and CDCl_3_. High‐resolution mass spectrometry analyses were performed using an Orbitrap Exploris 120 (Thermo Fisher Scientific) equipped with an electrospray ionization (ESI) source and a JMS‐700 (JEOL, Japan) equipped with a fast atom bombardment (FAB) source, respectively. UV/vis and photoluminescence spectra were obtained using a V‐730 spectrophotometer and FP‐8300 spectrofluorometer (JASCO, Inc.), respectively. The lowest unoccupied molecular orbital (LUMO) energy levels of π‐OSMs were determined from the bandgaps and HOMO energy levels using an FP‐8300 spectrofluorometer at excitation wavelengths of 391, 400, 401, and 409 nm. The thermal stabilities of π‐OSMs were determined using a Q200 differential scanning calorimeter (TA Instruments) and a TGA 4000 thermogravimetric analyzer (PerkinElmer, Inc.).

### Electrochemical Properties

The cyclic voltammograms of π‐OSMs were measured using a CHI650 B electrochemical analyzer (CH Instruments, Inc., TX, USA). The three‐electrode electrochemical cell was composed of a glassy carbon working electrode, a platinum counter electrode, and an Ag/Ag^+^ (0.01 m) reference electrode. The π‐OSMs (0.1 mm) and tetrabutylammonium hexafluorophosphate (TBAPF_6_, 0.1 m) in anhydrous tetrahydrofuran were prepared as a redox material and a supporting electrolyte, respectively. The ferrocene/ferrocenium (Fc/Fc^+^) redox couple was employed as the reference for all measurements.

### TE Properties Characterization of Hybrid and Neat Films

TE experiments were conducted based on the previous studies.^[^ ^13^, ^14^, ^15^ ^]^ To evaluate the *S* of the SWCNTs‐based TE samples, a custom‐built setup comprising of a temperature control unit, a measurement stage, and a TE measurement apparatus was used. The temperature differences between the hot and cold sides of the Peltier devices were controlled to achieve gradients of 8, 6, 4, and 2 °C. The *S* was measured using a Keithley 2700 multimeter. Open‐circuit voltage and short‐circuit current were acquired using a Keithley 2182A nanovoltmeter and a Keithley 6485 picoammeter. The temperature gradient of the Peltier devices was regulated using a Keithley 2604B SourceMeter. Square shapes (2 × 2 cm) of SWCNTs/π‐OSMs hybrid films were prepared by applying the films to Ag electrodes with a 1.5 cm gap between them. To estimate *S* and electrical resistivities, more than seven sets of SWCNTs/π‐OSMs film were fabricated. Raman studies were performed using a Renishaw inVia microscope (Renishaw Plc.) equipped with a 514 nm laser source. The thicknesses of TE films were determined using a Mini SEM JCM‐6000 (JEOL Ltd., Japan). The transport characteristics of the SWCNTs‐based samples were identified using the Van der Pauw method, which was performed by measuring the Hall effect and temperature‐dependent resistances using an Ecopia HMS 5500 instrument. Square‐shaped samples (1 × 1 cm) were prepared to measure Hall voltages under a magnetic field of 0.55 T and an electrical current of 5 mA. Morphological studies, including SEM and EDS mapping, were conducted using an FE‐SEM GeminiSEM 560 (Carl Zeiss GmbH) installed at the National Center for Inter‐university Research Facilities (NCIRF) at Seoul National University. The thermal diffusivities of both pristine SWCNTs and SWCNTs/π‐OSMs films were determined using a LaserPIT‐M2 instrument (Advance Riko, Inc.) in the in‐plane direction. A DSC 204 F1 Phoenix system (Netzsch‐Gerätebau GmbH) was employed to measure the specific heat capacities of both SWCNTs and SWCNT/π‐OSMs. UPS and XPS spectra of both SWCNTs and SWCNTs/π‐OSMs films were acquired using a K‐Alpha^+^ Spectrometer (Thermo Scientific Inc.), while UPS spectra of π‐OSMs thin films were recorded on an AXIS SUPRA (Kratos, U.K) instrument.

## Conflict of Interest

The authors declare no conflict of interest.

## Author Contributions

T.K. and J.J. contributed equally to this work. J.J. and J.‐I.H. initialized the project. T.K. and J.J. synthesized and characterized the compounds. T.K. designed and performed the majority of experiments under the guidance of J.‐I.H. J.J. conducted TE transport studies under the guidance of S.K. All authors contributed in drafting the manuscript.

## Supporting information

Supporting Information

## Data Availability

The data that support the findings of this study are available from the corresponding author upon reasonable request.
