# Supplementary material for: Molecular Engineering for Enhanced Thermoelectric Performance of Single‐Walled Carbon Nanotubes/π‐Conjugated Organic Small Molecule Hybrids
Source: Adv Sci (Weinh). 2023 Oct 20;10(33):2302922. doi: 10.1002/advs.202302922 (PMC10667833; doi:10.1002/advs.202302922)
Supplement: Supplementary file 1 — Supporting Information [file ADVS-10-2302922-s001.pdf]

ADVANCED SCIENCE
Open Access

Supporting Information

for *Adv. Sci.*, DOI 10.1002/advs.202302922

Molecular Engineering for Enhanced Thermoelectric Performance of Single-Walled Carbon Nanotubes/π-Conjugated Organic Small Molecule Hybrids

Tae-Hoon Kim, Jae Gyu Jang, Sung Hyun Kim and Jong-In Hong**

Supporting Information

Molecular engineering for enhanced thermoelectric performance of single-walled carbon nanotubes/π-conjugated organic small molecule hybrids

Tae-hoon Kim†, Jae Gyu Jang†, Sung Hyun Kim and Jong-In Hong**

Synthesis and Characterization

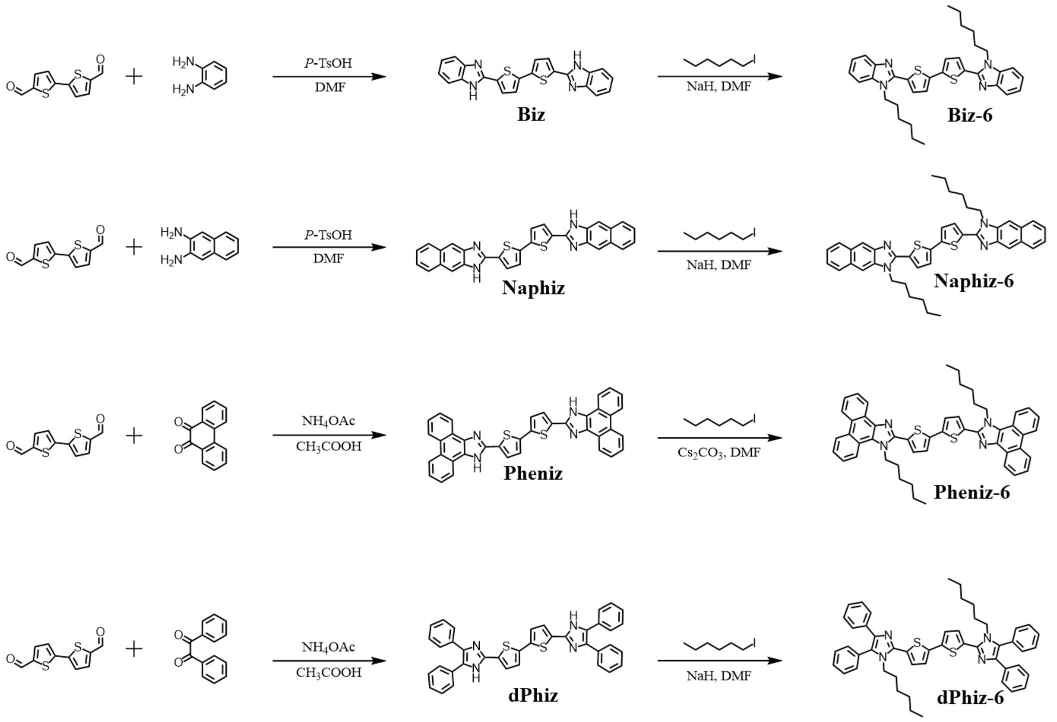

Scheme 1 Synthetic scheme of **Biz-6**, **Naphiz-6**, **Pheniz-6**, and **dPhiz-6**

Synthesis of 5,5'-[2,2'-bithiophene]-bis[1*H*-benzimidazole] (Biz)

To a stirred mixture of 2,2'-bithiophene-5,5'-dicarboxaldehyde (100 mg, 0.45 mmol) and *o*-phenylenediamine (146 mg, 1.35 mmol) in 5.0 ml of anhydrous DMF, *p*-toluenesulfonic acid (8.6 mg, 0.045 mmol) was added in one portion. The reaction mixture was stirred at 90 °C for 16 h. The resulting hot product mixture was directly poured into ice water, then cooled and precipitated. The precipitate was filtered and dried under an IR lamp to afford a

brown solid (193.5 mg, 100%). ^{1}H NMR (500 MHz, DMSO-d_6) δ 7.21 (4H, m), 7.52 (2H, d), 7.61 (2H, d), 7.80 (2H, d), 13.04 (2H, s). MS (MALDI-TOF) calculated for [M+H = $C_{22}H_{15}N_4S_2$]$^+$, calculated 399.073, found 399.053.

Synthesis 5,5'-[2,2'-bithiophene]-bis(1*H*-naphtho[2,3-*d*]imidazole) (Naphiz)

To a stirred mixture of 2,2'-bithiophene-5,5'-dicarboxaldehyde (99.4 mg, 0.44 mmol) and 2,3-diaminonaphthalene (176.9 mg, 1.12 mmol) in 5.0 ml of anhydrous DMF, *p*-toluenesulfonic acid (8.6 mg, 0.045 mmol) was added in one portion. The reaction mixture was stirred at 80 °C for 14 h. The resulting hot product mixture was directly poured into ice water, then cooled and precipitated. The precipitate was filtered and dried under an IR lamp to afford a brown solid (203.2 mg, 91 %). ^{1}H NMR (500 MHz, DMSO-d_6) δ 7.38 (4H, m), 7.53 (2H, d), 7.65 (4H, m), 7.98 (4H, m), 8.17 (2H, s), 13.08 (2H, s). MS (MALDI-TOF) calculated for [M+H = $C_{30}H_{19}N_4S_2$]$^+$, calculated 499.104, found 499.081.

Synthesis of 5,5'-[2,2'-bithiophene]-5,5'-diylbis[1*H*-phenanthroimidazole] (Pheniz)

To a stirred mixture of 2,2'-bithiophene-5,5'-dicarboxaldehyde (155.7 mg, 0.70 mmol) and 9,10-phenathrenequinone (437.6 mg, 2.11 mmol) in 9.0 ml of acetic acid, ammonium acetate (1187.9 mg, 15.42 mmol) was added in one portion. The reaction mixture was stirred at 80 °C for 14 h. The resulting hot product mixture was directly poured into ice water, then cooled and precipitated. The precipitate was filtered and washed with deionized water, then triturated in a mixture of acetone and ether (v/v = 1:1) to give an orange solid with a quantitative yield (556.4 mg, 100%). ^{1}H NMR (500 MHz, DMSO-d_6) δ 7.54–7.74 (8H, m), 7.92 (2H, d, J = 4.0 Hz) 8.05 (2H, d, J = 4.0 Hz), 8.43 (2H, d, J = 8.0 Hz), 8.50 (2H, d, J = 8.0 Hz), 8.84 (2H, d, J = 8.0 Hz), 8.88 (2H, d, J = 8.0 Hz), 9.91 (2H, s). MS (MALDI-TOF) calculated for [M+H = $C_{38}H_{23}N_4S_2$]$^+$, calculated 599.135, found 599.136.

Synthesis of 5,5'-[2,2'-bithiophenyl]bis[4,5-diphenyl-1*H*-imidazole] (dPhiz)

To a stirred mixture of 2,2'-bithiophene-5,5'-dicarboxaldehyde (96.5 mg, 0.43 mmol) and benzil (273.6 mg, 1.31 mmol) in 5.0 ml of acetic acid, ammonium acetate (1004 mg, 13.03 mmol) was added in one portion. The reaction mixture was stirred at 90 °C for 16 h. The resulting hot product mixture was directly poured into ice water, then cooled and precipitated. The precipitate was first filtered and washed with deionized water, then triturated in ether and filtered again. The isolated product was washed with ether to give a yellow solid (244.2 mg, 93 %). ^{1}H NMR (500 MHz, DMSO-d_6) δ 7.23 (2H, m), 7.30 (4H, m), 7.39 (4H, t, J = 3.5 Hz), 7.45–7.50 (12H, m), 7.62 (2H, m), 7.91 (2H, d, J = 7.5 Hz), 12.89 (2H, s). MS (MALDI-TOF) calculated for [M+H = $C_{38}H_{27}N_4S_2]^+$, calculated 603.167, found 603.251.

Synthesis of 5,5'-bis-(1-hexyl-1*H*-benzo[*d*]imidazole-2-yl])-2,2'-bithiophene (Biz-6)

5,5'-bis(^{1}*H*-benzo[d]imidazole-2-yl)-2,2'-bithiophene (173.9 mg, 0.43 mmol) and sodium hydride (57 mg, 1.43 mmol) in 1.5 ml anhydrous DMF was stirred at RT for 1 h, to which hexyl iodide (277.9 mg, 1.31 mmol) in 2.8 ml anhydrous DMF was added dropwise. The reaction mixture was vigorously stirred at 70 °C for 1.5 h. The crude product was concentrated in a high vacuum and purified by column chromatography with gradient elution of n-hexane and ether, then collected by eluting with THF. The concentrated product was recrystallized from chloroform using an excessive amount of n-hexane to afford a yellowish-orange solid (149.3 mg, 60 %). ^{1}H NMR (500 MHz, DMSO-d_6) δ 0.80 (6H, m), 1.23 (8H, m), 1.32 (4H, m), 1.76 (4H, t, J = 6.0 Hz), 4.50 (4H, t, J = 7.5 Hz), 7.26 (4H, dt, J = 23.5 Hz, J=7.5 Hz), 7.58 (2H, d, J = 3.9 Hz), 7.64 (4H, dd, J = 7.8 Hz, J = 4.0 Hz), 7.69 (2H, d, J = 4.0 Hz). ^{13}C NMR (125 MHz, DMSO-d_6) δ 14.22, 22.41, 26.18, 29.72, 31s.20, 44.56, 111.09, 119.35, 122.87, 123.31, 126.53, 128.85, 132.68, 136.72, 138.48, 142.82, 146.38. HRMS (ESI) [M+H = $C_{34}H_{39}N_4S_2]^+$, calculated 567.2610, found 567.2609.

Synthesis of 5,5'-bis-(1-hexyl-1*H*-naphtho[2,3-*d*]imidazole-2-yl])-2,2'-bithiophene (Naphiz-6)

5,5'-[2,2'-bithiophene]-bis(1*H*-naphtho[2,3-*d*]imidazole) (251.9 mg, 0.50 mmol) and sodium hydride (59.2 mg, 0.62 mmol) in 2.5 ml anhydrous DMF was stirred at RT for 1 h, to which hexyl iodide (201 mg, 0.95 mmol) in 3.5 ml of anhydrous DMF was added dropwise. The reaction mixture was vigorously stirred at 80 °C for 1.5 h. The crude product was concentrated in a high vacuum and purified by column chromatography with gradient elution of n-hexane and ether, then collected by eluting with chloroform. The concentrated product was recrystallized from chloroform using an excessive amount of n-hexane to afford a brown solid (149.1 mg, 32 %). ^{1}H NMR (500 MHz, $CDCl_3$) δ 0.91 (6H, t, *J* = 7.0 Hz), 1.37 (8H, m), 1.49 (4H, m), 1.96 (4H, p, *J* = 7.5 Hz), 4.42 (4H, t, *J* = 7.5 Hz), 7.37 (2H, d, *J* = 4.0 Hz), 7.41 (4H, ddd, *J* = 6.5 Hz, *J* = 4.0 Hz, *J* = 1.5 Hz), 7.54 (2H, d, *J* = 4.0 Hz), 7.67 (2H, s), 7.91 (2H, d, *J* = 7.5 Hz), 8.00 (2H, d *J* = 7.5 Hz), 8.26 (2H, s). ^{13}C NMR (125 MHz, $CDCl_3$) δ 13.94, 22.53, 26.67, 29.61, 31.40, 45.15, 105.27, 116.50, 123.59, 124.42, 125.41, 127.41, 128.47, 128.83, 130.69, 136.88, 139.89, 142.92, 150.32. HRMS (ESI) $[M+H = C_{42}H_{43}N_4S_2]^+$, calculated 667.2924, found 667.2925.

Synthesis of 5,5'-bis-(1-hexyl-1*H*-phenanthro[9,10-*d*]imidazole-2-yl)-2,2'-bithiophene (Pheniz-6)

5,5'-[2,2'-bithiophene]-5,5'-diylbis[1H-phenanthroimidazole] (210 mg, 0.35 mmol) and cesium carbonate (254 mg, 0.78 mmol) in 2.5 ml anhydrous DMF was stirred at RT for 1 h, to which hexyl iodide (201 mg, 0.95 mmol) in 3.5 ml of anhydrous DMF was added dropwise. The reaction mixture was vigorously stirred at 80 °C for 1.5 h. The crude product was concentrated in a high vacuum and purified by column chromatography with gradient

elution of n-hexane and ether, then collected by eluting with dichloromethane. The concentrated product was recrystallized from n-hexane to give a yellow solid (87.9 mg, 33 %). ^{1}H NMR (500 MHz, $CDCl_3$) δ 0.92 (6H, m), 1.38 (12H, m), 2.13 (4H, t, *J* = 12.0 Hz), 4.80 (4H, t, *J* = 13.5 Hz), 7.41 (2H, s), 7.64–7.72 (10H, m), 8.26 (2H, m), 8.69 (2H, d, *J*=8.0 Hz), 8.83 (4H, d). HRMS (FAB) [M+H = $C_{50}H_{47}N_4S_2$]$^+$, calculated 767.3242, found 767.3247.

Synthesis of 5,5’-bis-(1-hexyl-4,5-diphenyl-1*H*-imidazole-2-yl)-2,2’-bithiophene (dPhiz-6)

A mixture of 5,5’-[2,2’-bithiophenyl]bis[4,5-diphenyl-1H-imidazole] (207.0 mg, 0.34 mmol) and sodium hydride (42 mg, 1.03 mmol) in anhydrous DMF was stirred at RT for 1 h, to which hexyl iodide (230 mg, 1.03 mmol) in 3.5 ml of anhydrous DMF was added dropwise. The reaction mixture was vigorously stirred at 90 °C for 1 h and then allowed to cool slowly to RT to facilitate the formation of crystals. The crystal was filtered, washed successively with deionized water and n-hexane, and then dried using an IR lamp, yielding a yellowish-orange solid. Meanwhile, the unfiltered product was extracted with chloroform, washed with deionized water, and dried over Na_2SO_4. The resulting product was purified using silica gel chromatography with a gradient elution of n-hexane and ether, then collected by eluting with THF. The concentrated residue was recrystallized from chloroform using an excessive amount of n-hexane to afford a reddish-orange solid (combined yield: 223.9 mg, 85 %). ^{1}H NMR (500 MHz, $CDCl_3$) δ 0.80 (6H, t, 7.5 Hz), 1.14 (12H, m), 1.60 (4H, p, 7.5 Hz), 3.99 (4H, t, *J* = 7.5 Hz), 7.14 (2H, t, *J* = 7.0 Hz), 7.20 (4H, t, *J* = 7.5 Hz), 7.26 (2H, d, *J* = 7.5 Hz), 7.32 (2H, m), 7.40 (4H, m), 7.50 (10H, m). ^{13}C NMR (125 MHz, $CDCl_3$) δ 13.801, 22.250, 26.042, 30.419, 30.882, 44.887, 124.370, 126.429, 126.847, 128.032, 128.868, 129.035,

130.426, 131.155, 137.834, 140.790. HRMS (FAB) [M+H = $C_{50}H_{51}N_4S_2]^+$, calculated 771.3555, found 771.3553.

Determination of the adsorption amounts of π-OSMs

The physical adsorption amount of stilbene derivatives on SWCNTs was calculated via quantitative UV analysis using JASCO V-730 UV-visible spectrophotometer (JASCO, Inc.) as conducted in previous literature.[1-2]

$$\mathrm{wt}\,\% = \frac{m_{small\ molecule}}{m_{small\ moluecle} + m_{SWNT}}$$

Three solutions of different concentrations (5, 10, and 20 μM) of each OSM in anhydrous THF were prepared to obtain calibration curves of each OSM solution. An equal amount of SWCNTs (1 mg in 1 ml THF) was added into different amounts of OSMs in THF to prepare 20, 40, 60, and 80 wt% of composite solutions. Hybrid material films were fabricated by the vacuum filtration method using nylon membrane filters (see **Figure S11** for details). The corresponding absorbance of four unadsorbed π-OSMs at each concentration was recorded in UV-Vis spectrophotometer. Absorbances of unadsorbed π-OSM residues were automatically measured using the Beer-Lambert law. The unadsorbed amount of each π-OSM can be deduced from their molecular weights and molar concentrations acquired from the calibration curve. The actual adsorption amount of π-OSMs on SWCNTs/π-OSMs hybrid films can be calculated by subtracting the unadsorbed mass from the added mass of π-OSMs.

Supplementary figures and tables

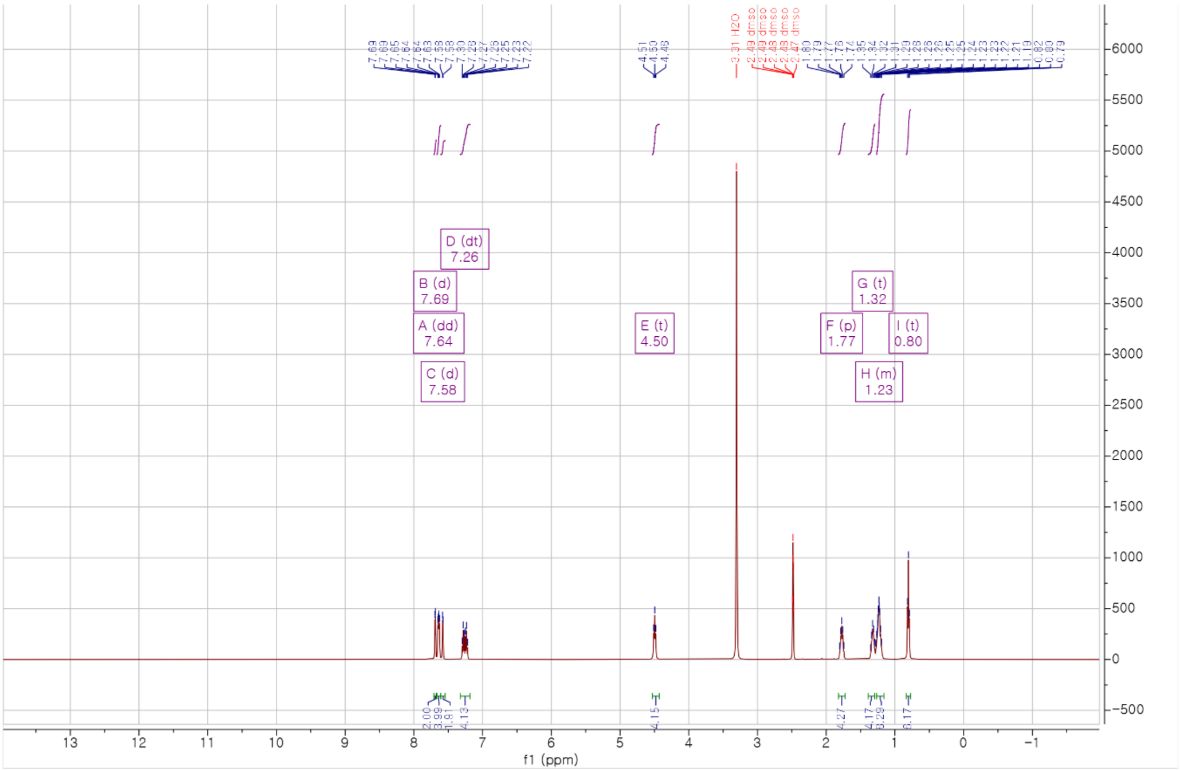

Figure S1. ^{1}H NMR spectrum of **Biz-6.**

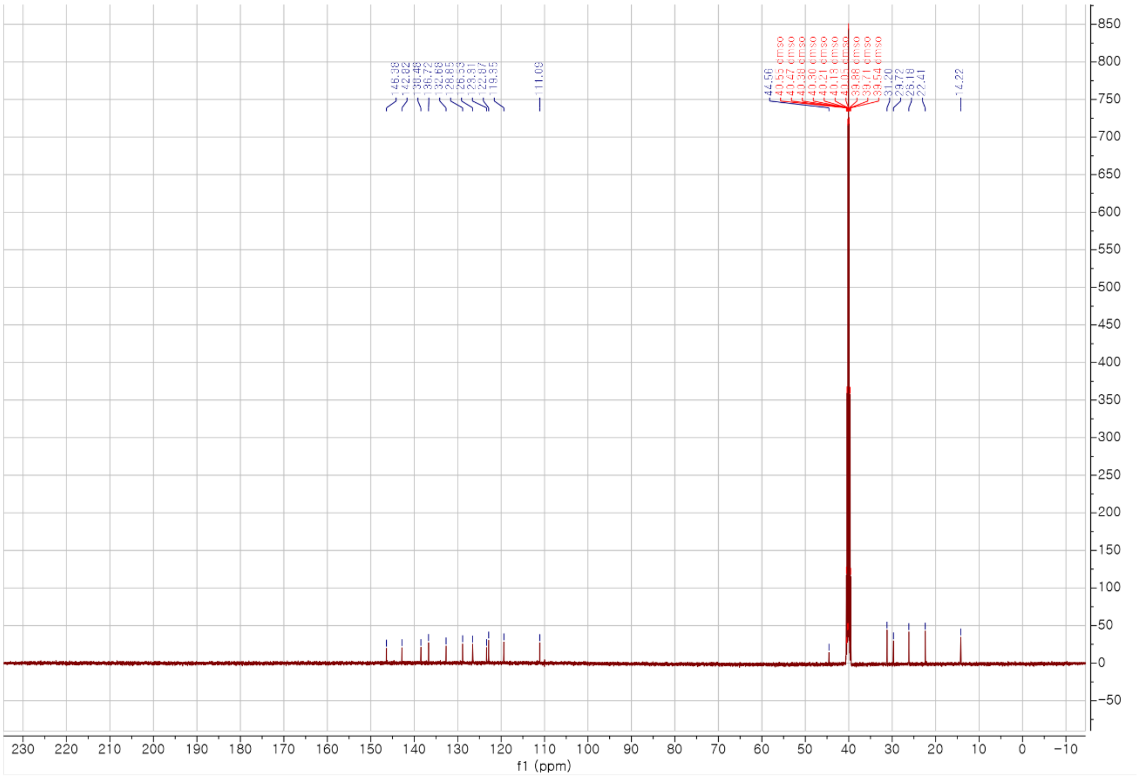

Figure S2 ^{13}C NMR spectrum of **Biz-6**.

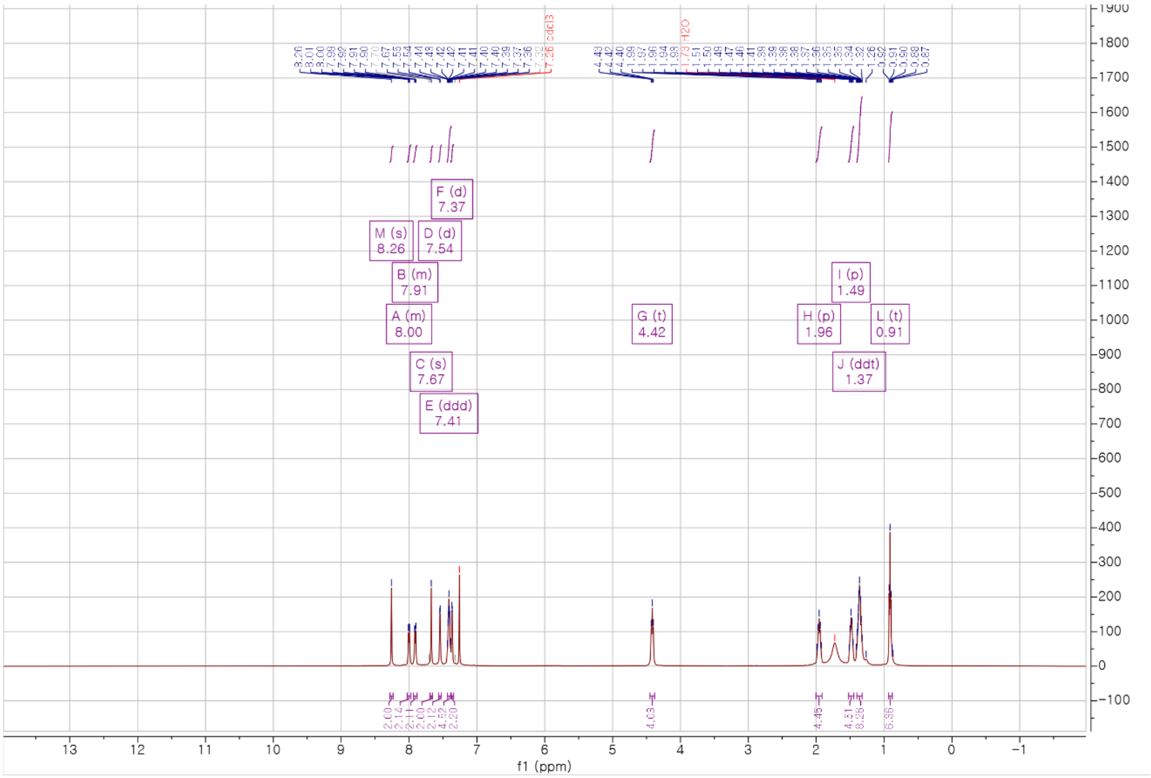

Figure S3. ^{1}H NMR spectrum of **Naphiz-6**.

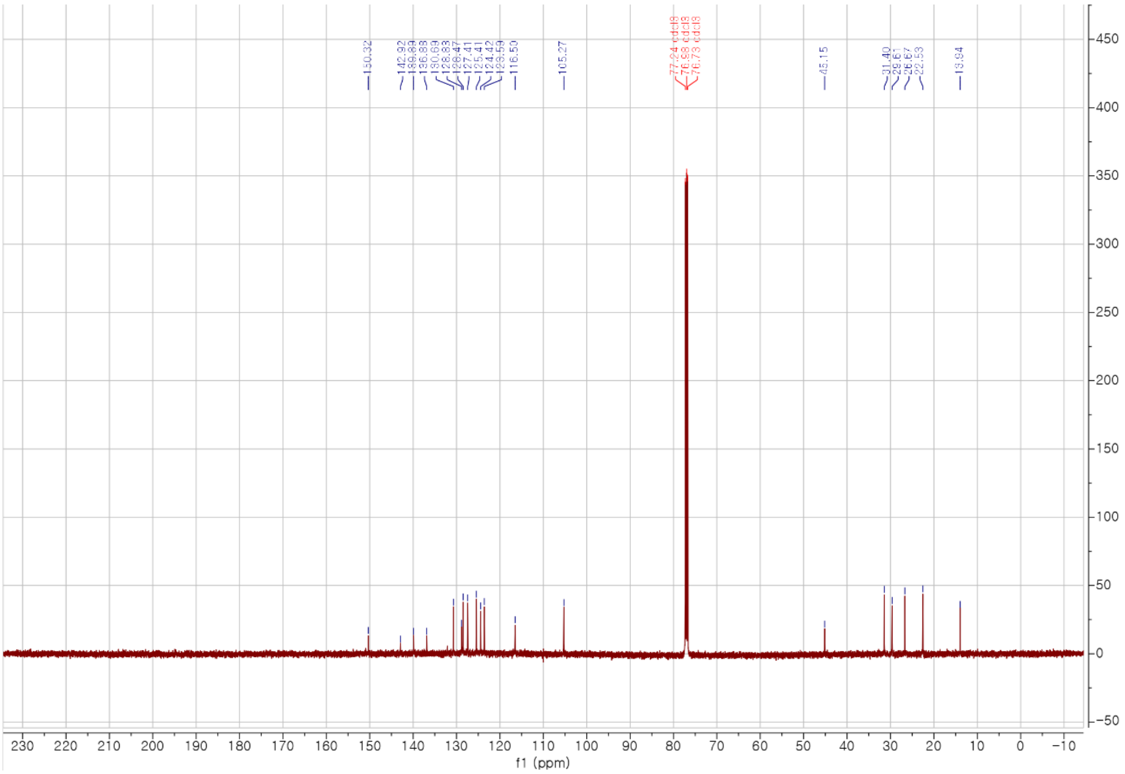

Figure S4. ^{13}C NMR spectrum of **Naphiz-6.**

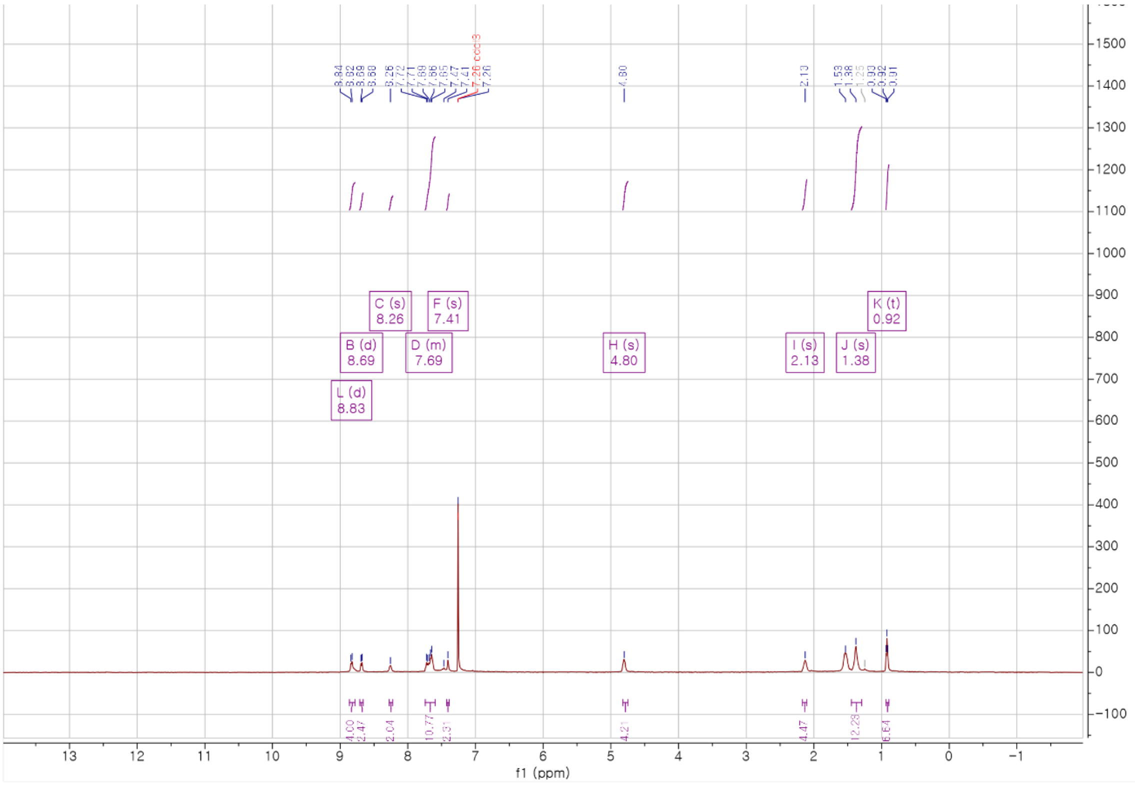

Figure S5. ^{1}H NMR spectrum of **Pheniz-6**.

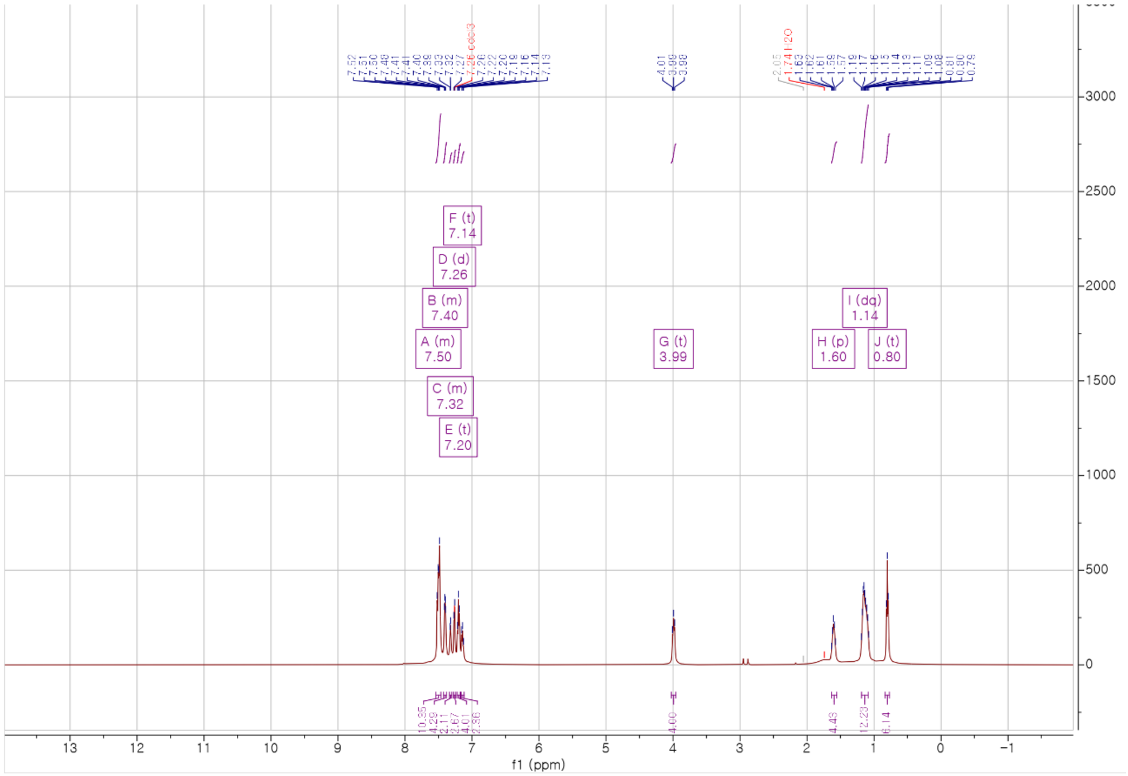

Figure S6. ^{1}H NMR spectrum of **dPhiz-6**.

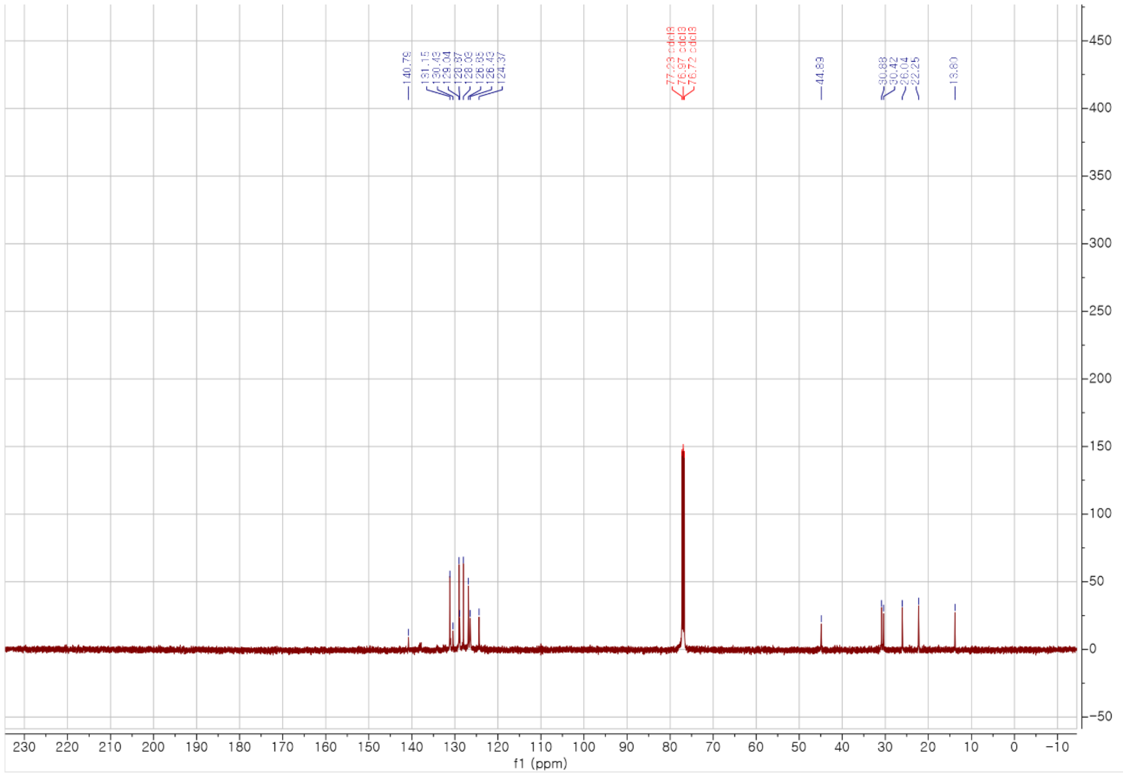

Figure S7. ^{13}C NMR spectrum of **dPhiz-6**.

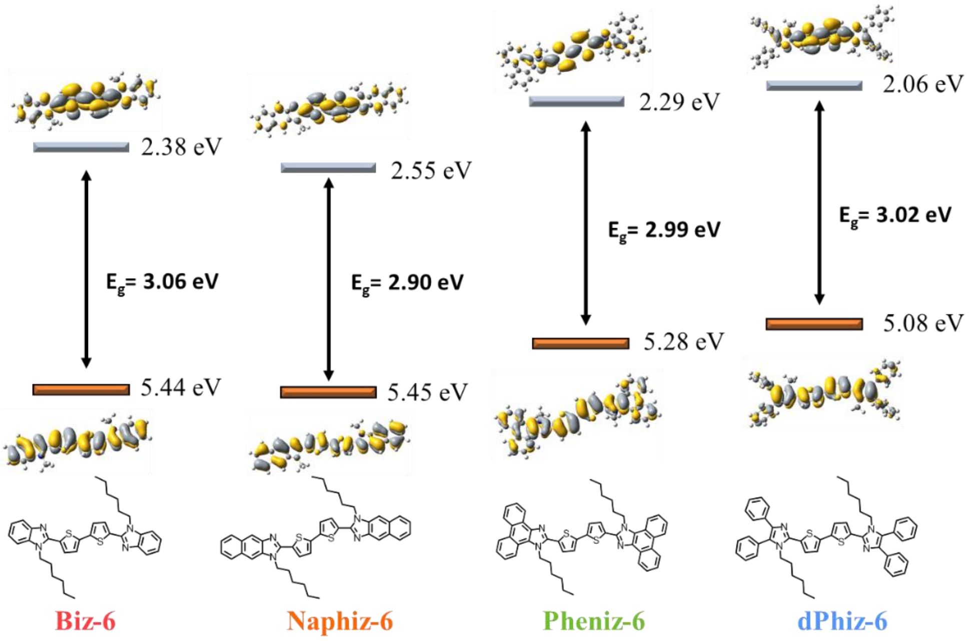

Figure S8. Calculated energy levels and molecular orbitals. DFT-minimized electronic levels and orbital distributions of **Biz-6**, **Naphiz-6**, **Pheniz-6**, **and dPhiz-6**.

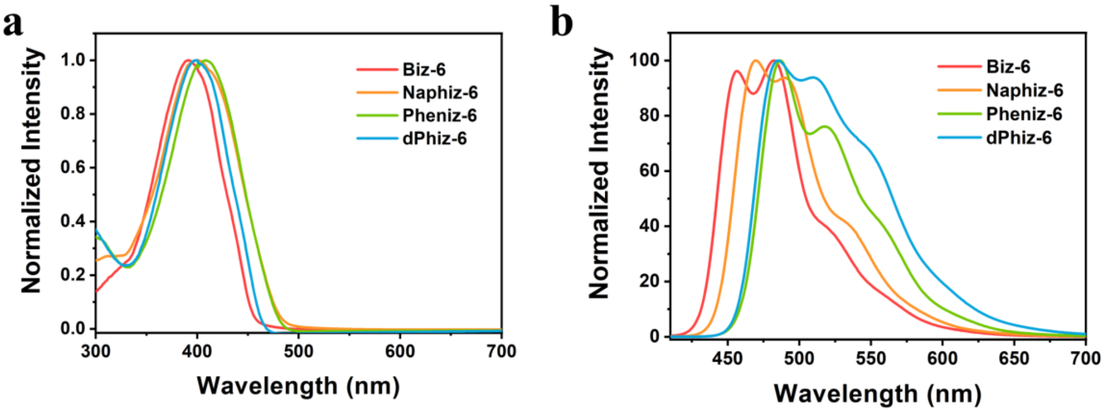

Figure S9. Optical spectra. a) UV/Vis and b) photoluminescence spectra of **Biz-6, Naphiz-6, Pheniz-6, and dPhiz-6.**

Table S1. Photophysical, electronic, and thermal properties of **Biz-6, Naphiz-6, Pheniz-6,** and **Pheniz-6.**

Compounds	$\lambda_{abs, max}$ [nm]	$\lambda_{em, max}$ [nm]	E_g^{opt} [eV]	E_g^{DFT} [eV]	$HOMO^{CV}$ [eV]	$HOMO^{DFT}$ [eV]	$LUMO^{DFT}$ [eV]	$LUMO^{Exp}$ [eV]	T_g, T_m, T_d [°C]
**Biz-6**	391	456, 482	2.62	3.06	-5.50	-5.44	-2.38	-2.88	- , 141, 372
**Naphiz-6**	400	469, 490	2.55	2.90	-5.53	-5.45	-2.55	-2.98	188, 230, 391
**Pheniz-6**	409	487, 518	2.59	2.99	-5.30	-5.28	-2.29	-2.71	225, 280, 428
**dPhiz-6**	399	485, 510	2.69	3.02	-5.29	-5.08	-2.06	-2.60	187, 225, 407

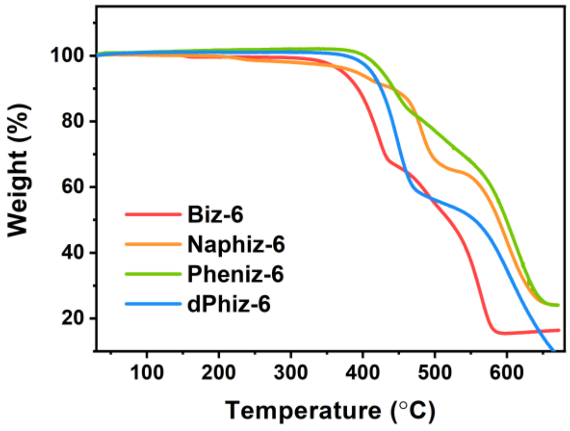

Figure S10. TGA curves of **Biz-6, Naphiz-6, Pheniz-6,** and **dPhiz-6**

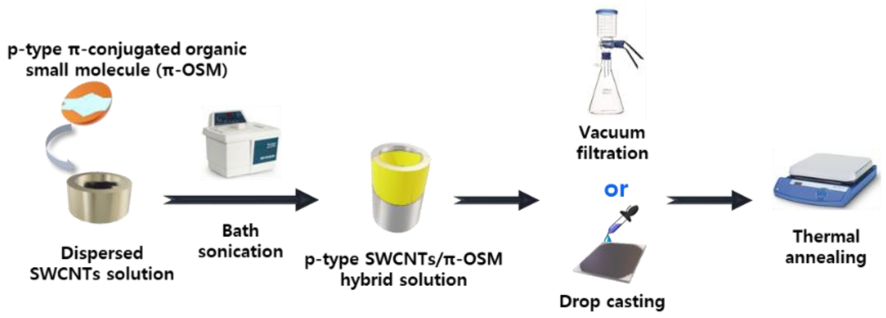

Figure S11. The fabrication procedure of SWCNTs/π-OSMs hybrid materials.

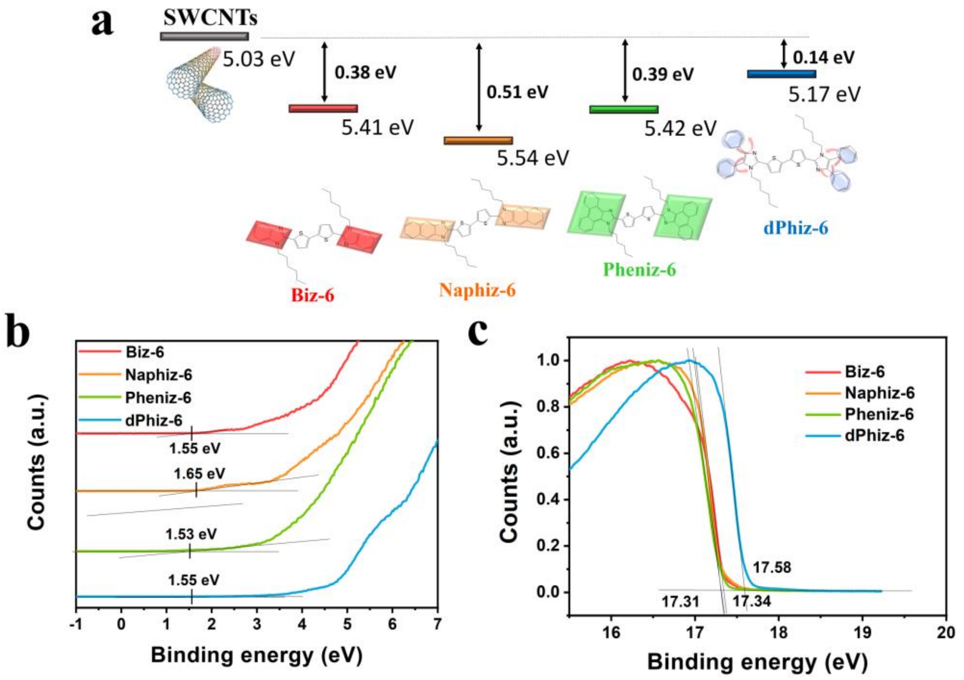

Figure S12. UPS spectra of π-OSM neat films. a) HOMO levels of SWCNTs, **Biz-6**, **Naphiz-6**, **Pheniz-6**, and **dPhiz-6**. Measured in the neat film states with b) secondary electron cutoffs and **c** valence band edge regions of **Biz-6**, **Naphiz-6**, **Pheniz-6**, and **dPhiz-6**, respectively.

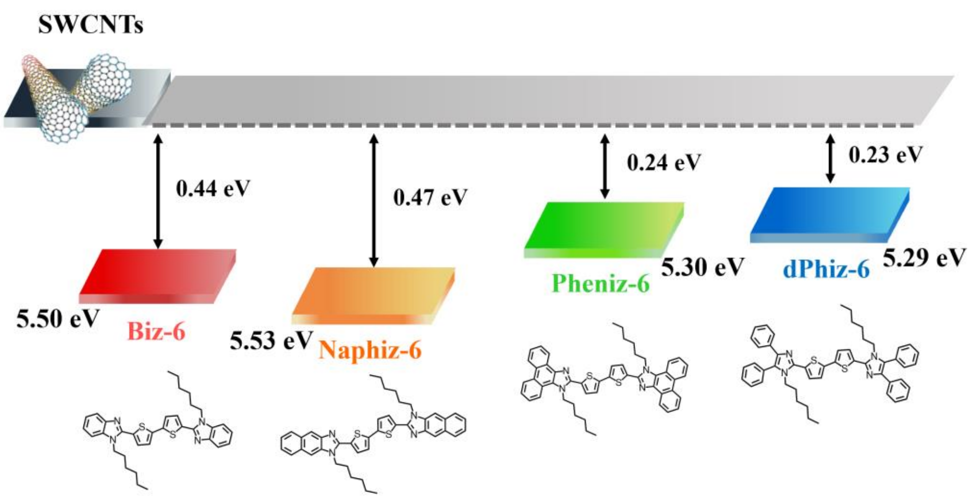

Figure S13. HOMO energy levels of **Biz-6**, **Naphiz-6**, **Pheniz-6**, and **dPhiz-6** obtained by cyclic voltammetry.

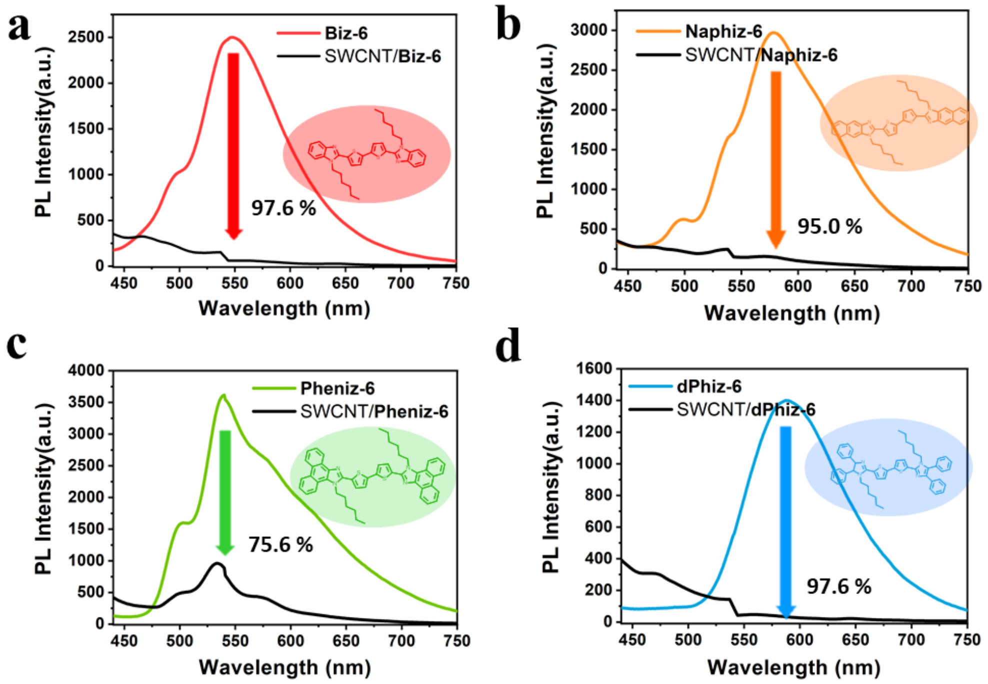

Figure S14. Photoluminescence spectra of SWCNTs/π-OSM hybrids. a) SWCNTs/**Biz-6**, b) SWCNTs/**Naphiz-6**, c) SWCNTs/**Pheniz-6,** and d) SWCNTs/**dPhiz-6** and their respective neat π-OSM films**.** Note that the casted mass of small molecules on neat and hybrid films are identical.

Table S2. Detailed adsorption ratios of SWCNTs/**Biz-6**, SWCNTs/**Naphiz-6**, SWCNTs/**Pheniz-6**, and SWCNTs/**dPhiz-6** hybrids.

Hybrid film	Added molecular	Adsorbed amount onto
SWCNTs/**Biz-6**	20	19.8
	40	23.3
	60	49.1
	80	62.3
SWCNTs/**Naphiz-6**	20	19.8
	40	29
	60	49
	80	57.9
SWCNTs/**Pheniz-6**	20	19.8
	40	22.3
	60	36.3
	80	50
SWCNTs/**dPhiz-6**	20	19.9
	40	32
	60	54.5
	80	65.3

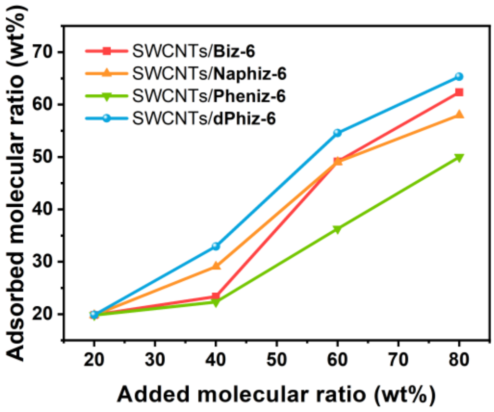

Figure S15. Adsorption patterns of SWCNTs/π-OSM hybrids. Adsorbed molecular ratios of SWCNTs/**Biz-6**, SWCNTs/**Naphiz-6**, SWCNTs/**Pheniz-6**, and SWCNTs/**dPhiz-6** hybrids.

Table S3. Thermoelectric characteristics for pristine SWCNTs and hybrid films at different molecular ratios.

Films (wt%)		Seebeck coefficient (μV K^{-1})	Electrical conductivity (S cm^{-1})	Power factor (μW m^{-1} K^{-2})
Pristine SWCNTs		60.9 ± 3.7	2209.6 ± 0.3	820.3 ± 3.1
SWCNTs/**Biz-6**	19.8	86.8 ± 2.4	1275.2 ± 13.3	905.5 ± 52.1
	23.3	87.5 ± 1.1	1243.8 ± 0.3	953.0 ± 26.1
	49.1	97.5 ± 3.3	637.6 ± 0.8	608.0 ± 41.8
	62.3	94.4 ± 3.9	580.1 ± 29.5	517.8 ± 43.8
SWCNTs/**Naphiz-6**	19.8	72.3 ± 0.5	1168.4 ± 0.5	625.8± 10.4
	29	77.6 ± 1.2	963.9 ± 0.2	581.7 ± 18.6
	49	79.4 ± 0.7	596.0 ± 41.8	291.2 ± 5.1
	57.9	74.6 ± 2.1	301.7 ± 23.0	207.0 ± 11.9
SWCNTs/**Pheniz-6**	19.8	73.9 ± 2.7	1099.3 ± 0.5	602.6 ± 44.1
	22.3	79.7 ± 3.9	851.8 ± 0.2	543.0 ± 54.0
	36.3	87.0 ± 3.8	461.5 ± 0.1	350.1 ± 30.5
	50	81.1 ± 2.9	314.6 ± 0.1	207.3 ± 15.0
SWCNTs/**dPhiz-6**	19.9	110.4 ± 2.6	1671.4 ± 0.2	2038.4 ± 97.0
	32	102.1 ± 5.9	1337.9 ± 0.1	1400.1 ± 160.5
	54.5	97.4 ± 1.5	701.6 ± 35.1	666.4 ± 21.7
	65.3	104.7 ± 5.4	564.0 ± 31.3	620.5 ± 64.4

Table S4. Thermoelectric properties of SWCNTs/**Biz**, SWCNTs/**Naphiz**, and SWCNTs/**dPhiz**.

Films (wt%)		Seebeck coefficient (μV K^{-1})	Electrical conductivity (S cm^{-1})	Power factor (μW m^{-1} K^{-2})
SWCNTs/**Biz**	20	41.6 ± 3.1	941.6 ± 24.5	164.6 ± 28.6
	40	42.6 ± 2.4	446.2 ± 17.5	81.4± 9.7
	60	46.7 ± 2.6	303.3 ± 33.7	66.8 ± 12.8
	80	39.9 ± 1.4	251.0 ± 19.2	40.3 ± 5.9
SWCNTs/**Naphiz**	20	39.9 ± 2.8	690.6 ± 49.1	111.8 ± 23.2
	40	43.5 ± 3.3	454.3 ± 22.5	86.7± 13.1
	60	46.7 ±2.9	240.0 ± 13.2	52.9 ± 9.5
	80	40.9 ± 1.1	155.7 ± 6.7	26.1 ± 2.1
SWCNTs/**dPhiz**	20	43.3 ± 1.7	1719.5 ± 270.7	320.1 ± 27.7
	40	47.5 ± 2.4	1265.8 ± 37.0	287.9 ± 36.5
	60	55.2 ± 2.5	914.6 ± 63.6	280.8 ± 38.1
	80	51.3 ± 2.2	666.3 ± 33.6	176.5 ± 24.1

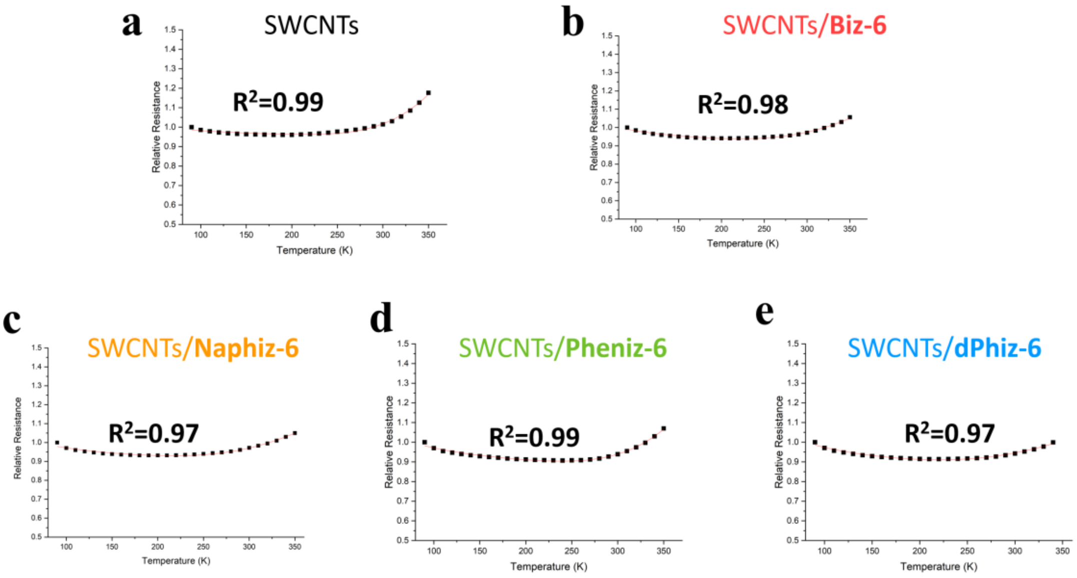

Figure S16. Fitted curves of temperature-dependent resistances in the temperature range of 90–350 K. (a) SWCNTs, (b) SWCNTs/**Biz-6**, (c) SWCNTs/**Naphiz-6**, (d) SWCNTs/**Pheniz-6**, (e) SWCNTs/**dPhiz-6**.

Table S5. Detailed fitting parameters. αR_m, βR_s, T_m, and T_0 values obtained by normalized temperature-dependent resistances of pristine SWCNTs, SWCNTs/**Biz-6**, SWCNTs/**Naphiz-6**, SWCNTs/**Pheniz-6,** and SWCNTs/**dPhiz-6** at 20 % molecular mixing ratio.

**Hybrid films**	**Fitting parameters**			
	αR_m	βR_s	T_m (K)	T_0 (K)
Pristine SWCNTs	335.5	0.89	2566.7	0.1
SWCNTs/**Biz-6** (20 wt%)	26.9	0.77	1838.8	1.2
SWCNTs/**Naphiz-6** (20 wt%)	25.2	0.77	1794.4	1.1
SWCNTs/**Pheniz-6** (20 wt%)	283.0	0.71	2549.0	3.12
SWCNTs/**dPhiz-6** (20 wt%)	9.8	0.69	1483.0	4.14

Table S6 Detailed transport parameters of pristine SWCNTs, SWCNTs/**Biz-6**, SWCNTs/**Naphiz-6**, SWCNTs/**Pheniz-6**, and SWCNTs/**dPhiz-6** evaluated by Hall-effect measurements (molecular mixing ratios of 20 and 40 wt%).

**Transport parameters**		Pristine SWCNTs	SWCNTs/ **Biz-6**	SWCNTs/ **Naphiz-6**	SWCNTs/ **Pheniz-6**	SWCNTs/ **dPhiz-6**
Electrical conductivity ($S\ cm^{-1}$)	20 wt%	2209.6 ± 0.3	1275.2 ± 13.3	1168.4 ± 0.5	1099.3 ± 0.5	1671.4 ± 0.2
	40 wt%		1243.8 ± 0.3	963.9 ± 0.2	851.8 ± 0.2	1337.9 ± 0.1
Carrier concentration ($10^{21}\ cm^{-3}$)	20 wt%	40.7 ± 4.7	27.7 ± 3.0	30.5 ± 4.9	31.1 ± 2.7	28.5 ± 0.9
	40 wt%		27.0 ± 3.9	29.3 ± 4.5	27.8 ± 2.2	24.5 ± 3.7
Carrier mobility ($10^{-2}\ cm^2\ V^{-1}\ s^{-1}$)	20 wt%	36.8 ± 4.2	29.1 ± 3.2	24.1 ± 4.1	22.1± 1.9	36.5 ± 1.2
	40 wt%		29.2 ± 4.2	21.0 ± 3.2	19.2 ± 1.6	34.8 ± 4.9
Hall's coefficient ($10^{-4}\ cm^3\ C^{-1}$)	20 wt%	1.5 ± 0.1	2.9 ± 0.3	2.1 ± 0.3	2.0 ± 0.1	1.9 ± 0.3
	40 wt%		2.5 ± 0.3	2.1 ± 0.3	2.2 ± 0.1	2.4 ± 0.5

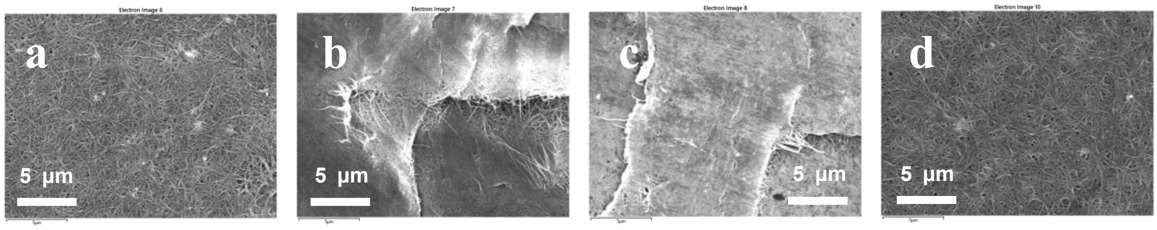

Figure S17. SEM images of (a) SWCNTs/**Biz-6 (**23 wt%), (b) SWCNTs/**Naphiz-6 (**29 wt%), (c) SWCNTs/**Pheniz-6 (**22 wt%), (d) SWCNTs/**dPhiz-6 (**32 wt%).

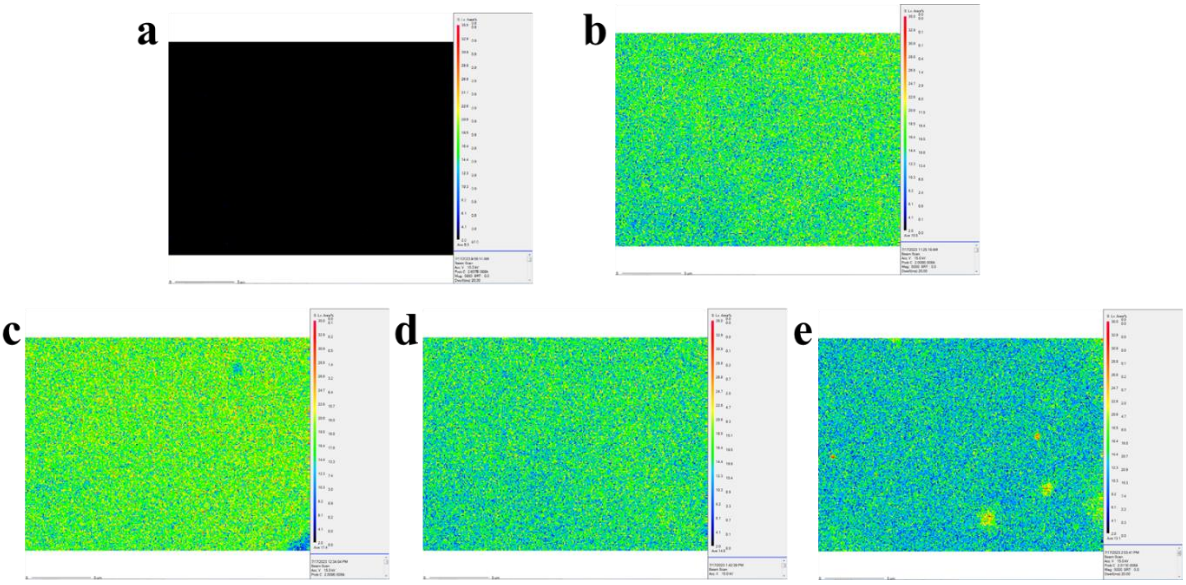

Figure S18. Electron probe microanalysis mapping images of Sulfur for (a) pristine SWCNTs, (b) SWCNTs/**Biz-6**, (c) SWCNTs/**Naphiz-6**, (d) SWCNTs/**Pheniz-6** (e) SWCNTs/**dPhiz-6** with a π-OSM mixing ratio of 80 wt%, respectively.

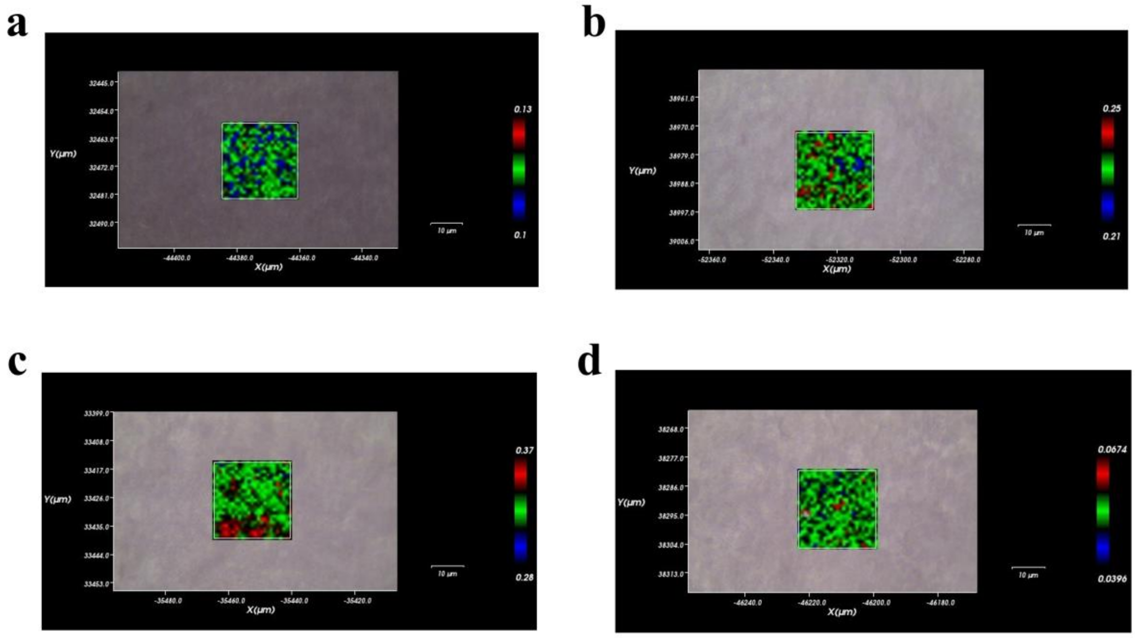

Figure S19. Raman mapping images of Sulfur for (a) SWCNTs/**Biz-6**, (b) SWCNTs/**Naphiz-6**, (c) SWCNTs/**Pheniz-6** (d) SWCNTs/**dPhiz-6** with a π-OSM mixing ratio of 80 wt%, respectively.

Table S7. Estimated thermal conductivities and *ZT* values. Densities (**d**), heat capacities ($\mathbf{C_p}$), thermal diffusivities (**α**), lattice thermal conductivities (κ_L), electronic thermal conductivities (κ_E), total thermal conductivities (κ_{total}), and *ZT* values of pristine SWCNTs and hybrid films at 20 wt% and 40 wt% molecular mixing ratios.

Thermoelectric materials	d (g cm^{-3})	C_p (J g^{-1} K^{-1})	α (10^{-6} m^2 s^{-1})	κ_L (W m^{-1} K^{-1})	κ_E (W m^{-1} K^{-1})	κ_{total} (W m^{-1} K^{-1})	ZT
Pristine SWCNTs	0.64	0.94	36.71	20.30	1.60	21.90	0.011±0.001
SWCNTs/**Biz-6**	0.34	0.93	39.06	11.58	0.57	12.15	0.022±0.001
SWCNTs/**Naphiz-6**	0.45	0.86	37.01	13.95	0.53	14.48	0.013±0.001
SWCNTs/**Pheniz-6**	0.37	0.91	28.6	9.02	0.49	9.63	0.019±0.001
SWCNTs/**dPhiz-6**	0.25	0.91	30.8	6.28	0.75	7.03	0.086±0.004
SWCNTs/**Biz-6***	0.13	0.97	28.44	3.03	0.56	3.59	0.079±0.002
SWCNTs/**Naphiz-6***	0.18	0.97	23.14	3.73	0.33	4.06	0.043±0.001
SWCNTs/**Pheniz-6***	0.19	0.99	32.48	5.89	0.38	6.27	0.026±0.003
SWCNTs/**dPhiz-6***	0.12	0.98	28.91	2.80	0.60	3.40	0.123±0.014

Table S8. Comparison of thermoelectric performances for carbon-nanotube-based organic hybrid materials.

*n-type hybrid materials.

Hybrid materials	Seebeck Coefficient (μV K^{-1})	Electrical Conductivity (S cm^{-1})	Power Factor (μW m^{-1} K^{-2})	ZT	Additional doping	References
SWCNTs/KOH/18-crown-6*	-33	2050	230	0.002 (310 K)	○	[3]
SWCNTs/ADTAb*	-44.5	642.4	124.4	0.0045	×	[4]
SWCNTs/ADLA4*	-60.7	529.6	195.2	0.006	×	[5]
SWCNTs/pyrene	44.9	978.6	197.5	0.0044	×	[6]
SWCNTs/flavin derivatives	59.4	176	62.1	0.0013	×	[7]
SWCNTs/F_4TCNQ doped-2	56.7	542.9	190.5	0.0196	○	[8]
SWCNTs/PEDOT:PSS	44.3	550	105	0.12	×	[9]
CSA-doped SWCNTs/PANI	65	769	176	0.12	○	[10]
CNT/PVAc	40-50	48	~12	0.006	×	[11]
$FeCl_3$-doped SWCNTs/P3HT	32.5	1000	107	0.015	○	[12]
As-grown CNT/PEI*	-69	3630	~1500	-	○	[13]
ICI doped CNT fibers	~7	~10000	~14000	0.007	○	[14]
SWCNTs/CzS	108.9	292	337.2	0.058	×	[1]
SWCNTs/Por-5F	53.3	982.4	279.3	-	×	[15]
n-PETT/CNT/PVC	30.2	429.3	39.1	~0.2 (330 K)	○	[16]
PCDTPT/FWCNT	~70	760	459	0.021	×	[17]
Doped CNT foam	32.6	4.02	0.43	7.6×10^{-4}	○	[18]
CNT/PTh	27.7	30	2.3	$\sim5\times10^{-4}$	×	[19]
SWCNTs/**dPhiz-6** (19 wt%)	113	1671.4	2135.5	0.091	×	This work
SWCNTs/**dPhiz-6** (32 wt%)	108	1337.9	1560.6	0.137	×	This work

SWCNTs/**dPhiz-6**	**0 day**	**1 day**	**9 days**	**12 days**	**22 days**
S/S_0	1	0.96	1.03	0.99	0.97
σ/σ_0	1	1.00	1.01	0.99	0.99
PF/PF_0	1	0.93	1.07	0.99	0.94

Table S9. TE stability of SWCNTs/**dPhiz-6** under the conditions of air exposure.

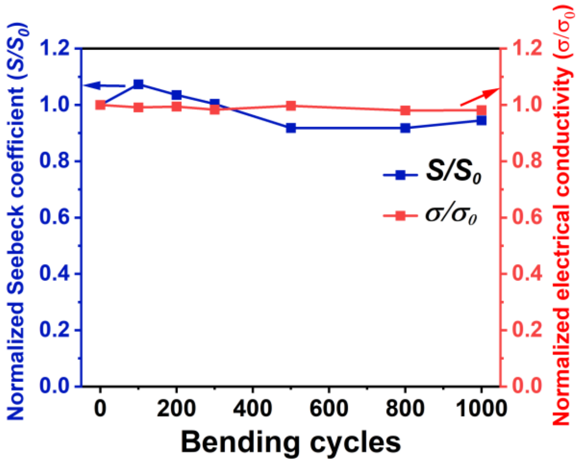

Figure S20. Normalized Seebeck coefficients (S/S_0) and electrical conductivities (σ/σ_0) of SWCNTs/**dPhiz-6** films as a function of the bending cycles.

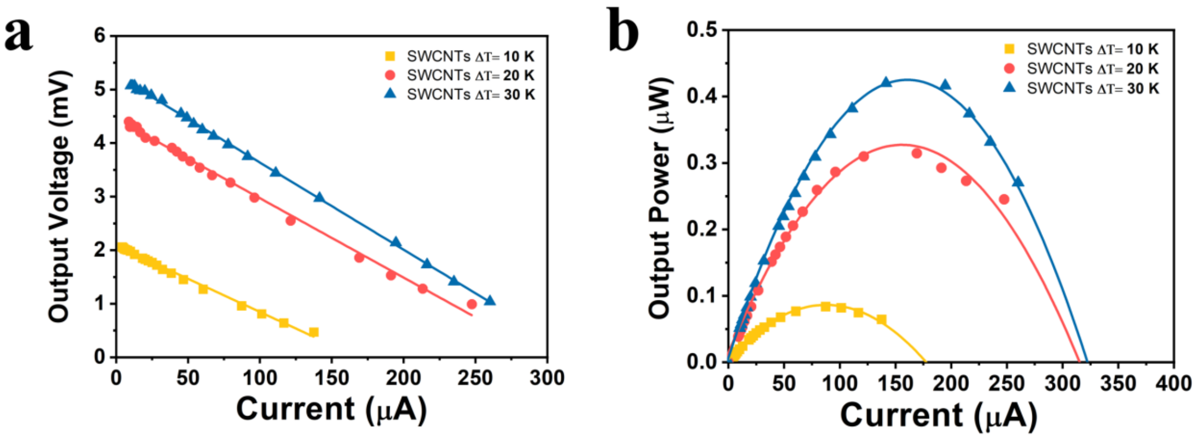

Figure S21. Voltage–current output of (a) pristine SWCNTs and power-current output curves of (b) pristine SWCNTs for thermoelectric modules at different temperature gradients.

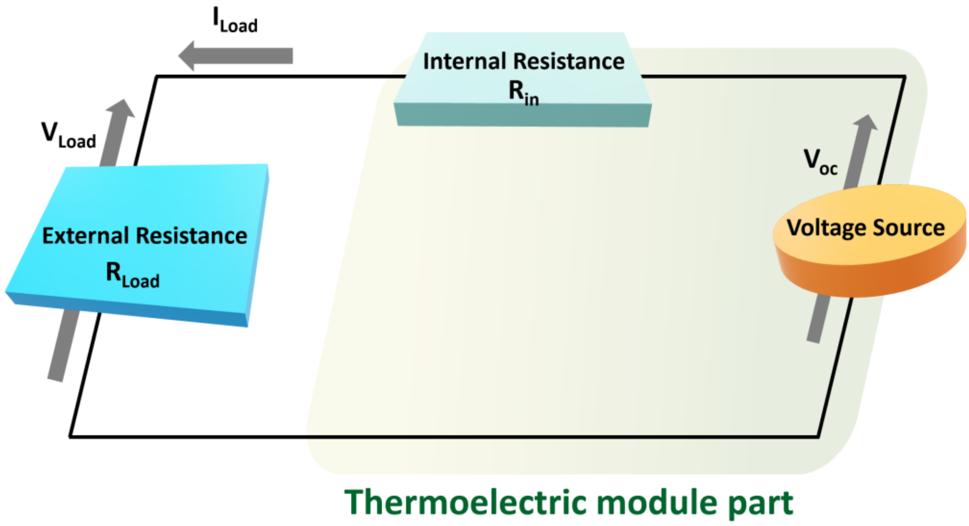

Figure S22. Schematic diagram of an electric circuit for a thermoelectric module connected to a load resistance.

References

[1] T.-h. Kim, J.-I. Hong, *ACS Appl. Mater. Interfaces* **2022**, *14*, 55627.
[2] T.-h. Kim, J. G. Jang, J.-I. Hong, *J. Mater. Chem. C* **2020**, *8*, 12795.
[3] Y. Nonoguchi, M. Nakano, T. Murayama, H. Hagino, S. Hama, K. Miyazaki, R. Matsubara, M. Nakamura, T. Kawai, *Adv. Funct. Mater.* **2016**, *26*, 3021.
[4] C. Gao, Y. Liu, Y. Gao, Y. Zhou, X. Zhou, X. Yin, C. Pan, C. Yang, H. Wang, G. Chen, L. Wang, *J. Mater. Chem. A* **2018**, *6*, 20161.
[5] Y. Liu, Q. Dai, Y. Zhou, B. Li, X. Mao, C. Gao, Y. Gao, C. Pan, Q. Jiang, Y. Wu, Y. Xie, L. Wang, *ACS Appl. Mater. Interfaces* **2019**, *11*, 29320.
[6] Y. H. Kang, Y.-C. Lee, C. Lee, S. Y. Cho, *Org. Electron.* **2018**, *57*, 165.
[7] W. Huang, F. Toshimitsu, K. Ozono, M. Matsumoto, A. Borah, Y. Motoishi, K. H. Park, J. W. Jang, T. Fujigaya, *Chem. Commun.* **2019**, *55*, 2636.
[8] J. G. Jang, S. Y. Woo, H. Lee, E. Lee, S. H. Kim, J.-I. Hong, *ACS Appl. Mater. Interfaces* **2020**, *12*, 51387.
[9] Q. Jiang, X. Lan, C. Liu, H. Shi, Z. Zhu, F. Zhao, J. Xu, F. Jiang, *Mater. Chem. Front.* **2018**, *2*, 679.
[10] Q. Yao, Q. Wang, L. Wang, L. Chen, *Energy Environ. Sci.* **2014**, *7*, 3801.
[11] C. Yu, Y. S. Kim, D. Kim, J. C. Grunlan, *Nano Lett.* **2008**, *8*, 4428.
[12] C. Bounioux, P. Díaz-Chao, M. Campoy-Quiles, M. S. Martín-González, A. R. Goñi, R. Yerushalmi-Rozen, C. Müller, *Energy Environ. Sci.* **2013**, *6*, 918.
[13] W. Zhou, Q. Fan, Q. Zhang, L. Cai, K. Li, X. Gu, F. Yang, N. Zhang, Y. Wang, H. Liu, W. Zhou, S. Xie, *Nat. Commun.* **2017**, *8*, 14886.
[14] N. Komatsu, Y. Ichinose, O. S. Dewey, L. W. Taylor, M. A. Trafford, Y. Yomogida, G. Wehmeyer, M. Pasquali, K. Yanagi, J. Kono, *Nat. Commun.* **2021**, *12*, 4931.
[15] Y. Zhou, X. Yin, Y. Liu, X. Zhou, T. Wan, S. Wang, C. Gao, L. Wang, *ACS Sustainable Chem. Eng.* **2019**, *7*, 11832.
[16] N. Toshima, K. Oshima, H. Anno, T. Nishinaka, S. Ichikawa, A. Iwata, Y. Shiraishi, *Adv. Mater.* **2015**, *27*, 2246.
[17] J. Jung, E. H. Suh, Y. J. Jeong, H. S. Yang, T. Lee, J. Jang, *ACS Appl. Mater. Interfaces* **2019**, *11*, 47330.
[18] M.-H. Lee, Y. H. Kang, J. Kim, Y. K. Lee, S. Y. Cho, *Adv. Energy Mater.* **2019**, *9*, 1900914.
[19] L. Wang, X. Jia, D. Wang, G. Zhu, J. Li, *Synth. Met.* **2013**, *181*, 79.
